# Demography of lemmings in response to changing snow conditions in the High Arctic

**DOI:** 10.1002/ecy.70216

**Published:** 2025-09-23

**Authors:** Mathilde Poirier, Gilles Gauthier, Florent Dominé, Dominique Fauteux

**Affiliations:** ^1^ Centre d'Études Nordiques Université Laval Québec City Québec Canada; ^2^ Department of Biology Université Laval Québec City Québec Canada; ^3^ Takuvik Joint International Laboratory Université Laval (Canada) and CNRS‐INSU (France) Québec City Québec Canada; ^4^ Department of Chemistry Université Laval Québec City Québec Canada; ^5^ Centre for Arctic Knowledge and Exploration Canadian Museum of Nature Ottawa Ontario Canada

**Keywords:** climate change, cycles, rain‐on‐snow, rodents, small mammals, snow ecology, winter

## Abstract

Changing snow conditions due to climate warming may negatively affect the northern fauna that depend on it for their winter survival. To avoid cold temperatures, Arctic lemmings seek refuge in areas with deep snowpack where they build nests in which they can reproduce if conditions are favorable. The presence of a soft depth hoar layer ensures efficient digging and facilitates lemming movement in the snow, but such favorable conditions are highly dependent on weather conditions at the beginning of winter. Using a 17‐year time series, we assessed the impact of snow conditions and specific weather events on lemming winter reproduction and population growth on Bylot Island in the Canadian High Arctic, a site characterized by a cold and dry Arctic climate. We focused on snow onset date, snow depth, and weather events leading to a hardening of the snow basal layer (i.e., rain‐on‐snow, melt‐freeze, and freezing rain) at the beginning of winter. We also examined possible differences between two lemming species, the brown lemming (*Lemmus trimucronatus*) and the collared lemming (*Dicrostonyx groenlandicus*), the latter presenting unique morphological adaptations to snowy environments. We found that the intensity of winter reproduction of both species was negatively related to the intensity of rain‐on‐snow, melt‐freeze, and freezing rain events. Winter population growth was also negatively related to the intensity of rain‐on‐snow and melt‐freeze events in brown lemmings but not in collared lemmings. Contrary to our expectation, no relationship was found between lemming demography and snow onset date or snow depth. We found a higher reproductive rate in collared than in brown lemmings, suggesting a more effective strategy to save energy for winter reproduction in the former species. Overall, this study shows that even moderate weather events, in comparison with other Nordic sites, can impact lemming population growth in winter, likely by reducing their capacity to reproduce due to a hardening of the snowpack. The expected increase in such weather events with climate change may threaten lemming populations even in the High Arctic, as well as predators that depend upon them.

## INTRODUCTION

Seasonality can produce temporary but essential habitats for many species that evolved with its recurrent climatic pattern. For instance, vernal pools created during rainy seasons support the development of numerous organisms, including insects and amphibians (Zedler, 2003). Similarly, in northern environments, the formation of an annual snowpack creates a temporary habitat for a multitude of resident animals, such as brown bears (*Ursus arctos*), willow ptarmigans (*Lagopus lagopus*), wolverines (*Gulo gulo*), and several small mammals (Andreev, 1991; Glass et al., 2021; Reid et al., 2012; Sorum et al., 2019). For animals living under the snowpack, the insulating properties of snow make it a good shelter against cold and also offer protection against predators (Bilodeau et al., 2013a; Duchesne et al., 2011a; Hansson & Henttonen, 1985). Nevertheless, physical characteristics of the snow can change drastically from year to year depending on annual weather conditions as well as in the long term in response to climate warming (Liston & Hiemstra, 2011; Mudryk et al., 2020), and this can have a profound impact on animals using this habitat.

Lemmings are small mammals that have adapted to the long and harsh Arctic winter by living within the snowpack for up to 9 months. They seek a deep snowpack to build their nests made of dry vegetation, which provides warmth and can even support reproduction under the right conditions (Duchesne et al., 2011a; Poirier et al., 2023; Reid et al., 2012). Their capacity to reproduce under the snowpack allows for rapid population growth during the winter period. To find food or mate, lemmings dig a network of tunnels in the softest snow layer, called the depth hoar, typically located at the base of the snowpack (Poirier et al., 2019; Sturm & Benson, 1997). Their ability to thrive in this habitat is a key factor allowing for the persistence of their populations and can explain why they have become a vital prey for most tundra predators (Schmidt et al., 2012; Therrien et al., 2014). Many lemming populations also go through large amplitude, multi‐annual cyclic fluctuations, mostly driven by density‐dependent changes in predator pressure (Bergeron et al., 2025; Fauteux et al., 2016; Gilg et al., 2003). However, recent literature highlighted the role of other factors, including snow properties, in affecting and in some cases dampening these cyclic fluctuations (Bilodeau et al., 2013b; Domine, Gauthier, et al., 2018; Gauthier et al., 2024; Kausrud et al., 2008).

The changing climate will inevitably modify characteristics of the snowpack, particularly in the Arctic where temperatures have already increased almost four times faster than the global average (Rantanen et al., 2022). As a result, delayed snow onset and increased precipitation are anticipated (Liston & Hiemstra, 2011). Moreover, greater temperature variations around the freezing point after the onset of the snowpack will increase the risk of liquid water formation due to melting in the snowpack, or to an input of liquid water during rain‐on‐snow events (Liston & Hiemstra, 2011). Subsequent refreezing of this water forms hard melt‐freeze layers within the snowpack, with rain‐on‐snow events forming the hardest and thickest refrozen layers (Domine, Gauthier, et al., 2018). When occurring at the beginning of winter, these melt‐freeze layers compromise the formation of a soft depth hoar layer (Domine et al., 2009), which can have a long‐lasting effect and be detrimental to lemmings (Berteaux et al., 2017; Domine, Gauthier, et al., 2018). Freezing rain can also lead to glazed ice on top of the snow and, although it is typically rare in the High Arctic (Roberts & Stewart, 2008), it could become more frequent with global warming (Groisman et al., 2016).

Hardening of the snowpack, mainly induced by rain‐on‐snow events, has been shown to threaten populations of herbivores such as caribous (*Rangifer tarandus*), rock ptarmigans (*Lagopus muta*), and east European voles (*Microtus levis*) (Hansen et al., 2013; Stien et al., 2012). In addition to hampering animal movements through the snow (Poirier et al., 2021), severe rain‐on‐snow events can encapsulate vegetation and deprive animals of their food source (Hansen et al., 2014; Sokolov et al., 2016). The fading out of small mammal population fluctuations and persistently low population abundances in some regions of the Arctic has been linked to changes in the snow regime, such as an increase in snow hardness (Cornulier et al., 2013; Hörnfeldt et al., 2005; Ims & Fuglei, 2005; Kausrud et al., 2008).

Winter reproduction is considered a crucial factor in lemming population outbreaks (Ims et al., 2011; Millar, 2001), but snow characteristics can modulate their ability to reproduce (Domine, Gauthier, et al., 2018). Indeed, in the presence of hard snow, lemmings increase their digging efforts (Poirier et al., 2021), which potentially reduces the energy available for reproduction. However, all lemming species may not be affected equally. For instance, the collared lemming (*Dicrostonyx groenlandicus*) appears to be better adapted to life in the snow than the brown lemming (*Lemmus trimucronatus*) with its white fur color, the growth of large bifid claws in winter, and its overall better performance when digging in hard snow (Hansen, 1957; Poirier et al., 2021; Zimova et al., 2018).

In this study, we assessed the impact of snow conditions and specific weather events on lemming winter demography under the cold and dry climate of the High Arctic. First, we hypothesized that lemming exposure to predation and thermoregulatory costs should increase with a delayed or shallow snow cover (Gilg et al., 2009). Accordingly, we predicted that a late snow onset and a shallow snow accumulation at the beginning of winter should reduce lemming winter reproduction and population growth. Second, we hypothesized that a hard basal snow layer induced by severe weather events increases lemming energetic costs to dig (Poirier et al., 2021) and negatively impacts their demography. Therefore, we predicted that rain‐on‐snow, melt‐freeze, and freezing rain events occurring at the beginning of winter should also reduce lemming winter reproduction and population growth. Third, we hypothesized that the effects of weather events on lemming winter demography should be exacerbated (1) by the presence of ermines (*Mustela richardsonii*), the only predator that can efficiently hunt lemmings under the snow (Bilodeau et al., 2013a), and (2) at high lemming density due to increased competition for food. Finally, we hypothesized that demographic responses to snow conditions and weather events will differ between lemming species due to differential adaptation to the winter environment. More specifically, winter reproduction and population growth of collared lemmings should be less impacted by hard snow compared to brown lemmings.

## METHOD

### Study area

The study took place in the Qarlikturvik Valley of Bylot Island (73°08′ N, 80°00′ W) between 2004 and 2022. This site is characterized by a cold and dry High Arctic climate with a mean temperature of −37°C during the coldest month in February and a mean snow depth of 31 cm in flat terrains at the end of winter (Domine et al., 2021a, 2021b). Snow usually sets in early October and ends in early June. The 51 km^2^ study area is mainly characterized by a mosaic of mesic, wetland, and riparian habitats. The mesic habitat is dominated by herbaceous plants, prostrate shrubs, and mosses (Audet et al., 2007), whereas the wetland habitat is dominated by mosses and graminoids (Gauthier et al., 2011). Despite these differences in vegetation, both habitats experience similar snow conditions (Poirier et al., 2023). The riparian habitat is defined as the tundra next to streams, including the slopes on either side, which favors deep snow accumulation (i.e., snowdrifts) due to wind deposition. Vegetation in the riparian habitat is a mix of mesic and wetland, following an increasing humidity gradient from the top to the bottom of slopes (Poirier et al., 2023).

### Lemming demographic parameters

We determined the intensity of lemming winter reproduction annually between 2007 and 2022 from winter nests sampled along forty 500‐m‐long permanent transects, equally and randomly distributed in the mesic and riparian habitats. The wetland habitat was not sampled since it is rarely used by lemmings in winter, likely due to the low quality of the snow cover (Poirier et al., 2023). Soon after snow melt, we slowly walked each transect and dissected every nest detected. We identified the species that occupied it (brown or collared) based on the size, shape, and color of feces (MacLean et al., 1974), a reliable method (Soininen et al., 2015). We also noted the occurrence of reproduction, which was determined when at least one third of all feces present were of distinctively small size, an indication that juveniles occupied the nest (Duchesne et al., 2011b). When feces of both species were found in the nest (about 5% of the time), we duplicated the nest in our dataset and considered it as a double occupation. When reproduction was detected in these nests, the layering order of feces indicated which species had reproduced. We estimated the proportion of reproductive nests by dividing the number of nests with reproduction by the total number of nests found each year separately for each species. In years of low lemming density, we could not find enough nests along the transects to accurately estimate proportions, so we included in the analysis nests found opportunistically in mesic and riparian habitats. Observers found nests opportunistically while conducting other activities in the field. Due to COVID‐19 travel restrictions, we could not collect nests in 2020 and 2021.

We obtained summer lemming densities from live trapping in two 11‐ha square grids located in mesic and wet habitats (one each). Each grid had 144 Longworth traps (100 from 2004 to 2006) spaced out at 30‐m intervals. Traps were baited with a small piece of apple and peanut butter, and stuffing was added to help animals keep their warmth. Trapping was performed in mid‐June, mid‐July, and mid‐August between 2004 and 2022, but not in 2020 and only in August 2021 due to COVID‐19. Traps were checked twice a day for three or four consecutive days during each trapping session. All animals trapped were identified to species, marked, and released (see Fauteux et al., 2015 for details). We obtained density estimates from spatially explicit capture–recapture models using the *secr* package version 4.6.0 (Efford, 2023) implemented in the R software version 4.1.0 (R Core Team, 2021) for each species, grid, and trapping session. We obtained species‐specific densities for each grid and for each month (June, July, and August).

We determined winter growth of lemmings in each grid from the natural log of the ratio between density in June and density in August of the previous year. Each winter is referred to by the year when it ended (e.g., winter 2003–2004 is referred to as winter 2004). Due to some zero values, we added 0.01 to all densities (lemmings/ha), which is half the smallest value (0.02) that could be determined on our trapping grids, that is, if only one individual was captured. In 2010, we used mean June–July lemming density because persistent snow led to an underestimation of lemming density in June. For winter 2004, density in August of the previous year (2003) was estimated from snap trapping data (Gruyer et al., 2008) based on the equations of Fauteux et al. (2018) because live trapping only started in 2004.

### Snow and weather data

Since 1993, an automated weather station located in our study area (BYLCAMP) records several snow and weather variables such as snow depth, air temperature, relative humidity, and wind velocity at hourly intervals (CEN, 2024). We obtained mean snow depth during the month of November with an SR50 acoustic gauge. In 2010 and between 2014 and 2017, no snow depth data were recorded due to a malfunction of the gauge. For 2014–2017, we used environmental data from another automated weather station located 1.7 km away. We adjusted data between the two stations using the 2018 and 2019 data, which were available at both stations (average snow depth difference: 0.83 cm, *R* ^2^ = 0.92 for the relationship of snow depth between the two stations).

The snow onset date corresponds to the first date of the season when snow covered more than 80% of our study area without returning below 50% cover at a later date during the season. To determine this value, we first obtained an interval of snow onset dates using MODIS images (Moderate Resolution Imaging Spectroradiometer; extracted from https://modis.gsfc.nasa.gov/). This satellite provides one image daily (500 m resolution) in which we detected the presence of snow on the ground with the normalized difference snow index (NDSI; Riggs & Hall, 2015), allowing us to calculate a percentage of snow cover over the study area (see Appendix S1 for details). However, images were often not usable due to the presence of clouds, leading to data gaps of up to 18 days. Having a more accurate snow onset date was crucial because it influences the partial melting processes we wished to determine. We refined snow onset dates by using other methods including photos of the study area taken daily by automated cameras (from 2016 to 2021 only) and hourly weather data recorded at our study site and in Mittimatalik (Pond Inlet), a town located about 90 km from our study site (see Appendix S1: Figures S1–S22). In Appendix S1: Table S1, we summarize how data sources were combined to determine the snow onset date each year.

To evaluate the impact of rain‐on‐snow, melt‐freeze, and freezing rain on lemming demography, we scored the occurrence and intensity of each of these weather events. We assumed that snow hardness increases linearly with the intensity of these weather events until it reaches a saturation point where the snow becomes ice. Scores were calculated between the snow onset date and November 30 because we were interested in weather events occurring at the beginning of winter when most of the depth hoar is formed (Domine, Belke‐Brea, et al., 2018). The rain‐on‐snow score was obtained by summing the number of hours with positive temperatures and a relative humidity >95%. Under these conditions, it is safe to assume that precipitations were most likely rain rather than snow, as the precipitation phase is a function of relative humidity. The melt‐freeze score was obtained by summing the number of hours with positive temperatures and a relative humidity <95%. Chances of liquid precipitation are less than 50% below this humidity level, and 0°C is the temperature at which snow grains start thawing and forming clusters when refreezing (Colbeck, 1982). Finally, we based our freezing rain score on categorical freezing rain reanalysis data, which estimate the presence/absence of freezing rain events every 6 h (NCEP North American Regional Reanalysis [NARR], extracted from https://psl.noaa.gov/). This reanalysis model combines observational data, satellite data, and output from numerical weather prediction models to estimate atmospheric conditions that favor the occurrence of freezing rain during a 6‐h period with a 32 km resolution. We summed the number of freezing rain events detected within a radius of 80 km from the center of our study site and multiplied this value by 6 to obtain hourly scores.

Between 2014 and 2022, we also measured snow basal density (i.e., the lowest 5 cm of the snowpack) in riparian and mesic habitat with a box cutter (Conger & McClung, 2009). These measurements were performed in May before snow melt, except in 2016 when no sampling was conducted due to logistical constraints. Each year, snow basal density measurements were taken at 1–11 snow pits dug in sites representative of each habitat over a 20 km^2^ area.

### Statistical analysis

We assessed the influence of snow conditions (snow onset, snow depth in November) and weather events (rain‐on‐snow, melt‐freeze, freezing rain) on the proportion of winter nests with reproduction using generalized linear models with a logit link, binomial distribution, and quasi likelihood methods for parameter estimation to account for overdispersion problems (glm function, Venables & Ripley, 2002). Lemming species, density dependence, and ermine abundance were added to the models as additive or interactive terms. We used lemming density in August of the previous year to test for density dependence and index of abundance obtained during the preceding summer for the ermine (data obtained from Bolduc et al., 2023). Because we previously found a nonlinear relation between lemming digging performance and snow hardness (Poirier et al., 2021), we log‐transformed weather event variables to determine whether they could provide a better model fit. We used the adjusted *R* ^2^ (calculated from the function *adjR2* of the *glmtoolbox* package; Vanegas et al. (2024)) as it evaluates the proportion of variance explained by the model (1 − deviance/deviance of the null model) while adjusting for the number of predictors.

We assessed the effect of snow conditions and weather events on winter growth of lemming populations using linear mixed‐effects models. Lemming species, density dependence, and ermine abundance were added to the models as additive or interactive terms as previously described. We used grid and year as crossed random effects. We evaluated the fit of the models using the amount of variation explained by fixed factors (*R* ^2^_*m*_) and by both fixed and random factors (*R* ^2^_*c*_) with the *MuMIn* package version 1.47.1 (Barton, 2022) following Nakagawa and Schielzeth (2013).

The evidence for a relationship was determined with the 95% CIs around the slope parameters, and robust SEs were used in linear models with unbalanced variance of residuals (clubSandwich package version 0.5.11; Pustejovsky, 2024). We used Cook's distance to evaluate the presence of influential data points in our analyses. When the Cook's distance exceeded the threshold of 4/*n*, where *n* is the sample size, we considered these data points as moderately influential (Bollen & Jackman, 1990), and we repeated the analyses without those points. To report effect size in the results, we calculated the percentage change over the entire range of data based on predicted values derived from the models.

As a complementary analysis, we examined the direct effect of snow basal density on winter reproduction and population growth using binomial and linear models, respectively. We could not compare these analyses with the previous ones due to the shorter time series (2014–2021).

## RESULTS

### Occurrence of specific weather events

**FIGURE 1:**
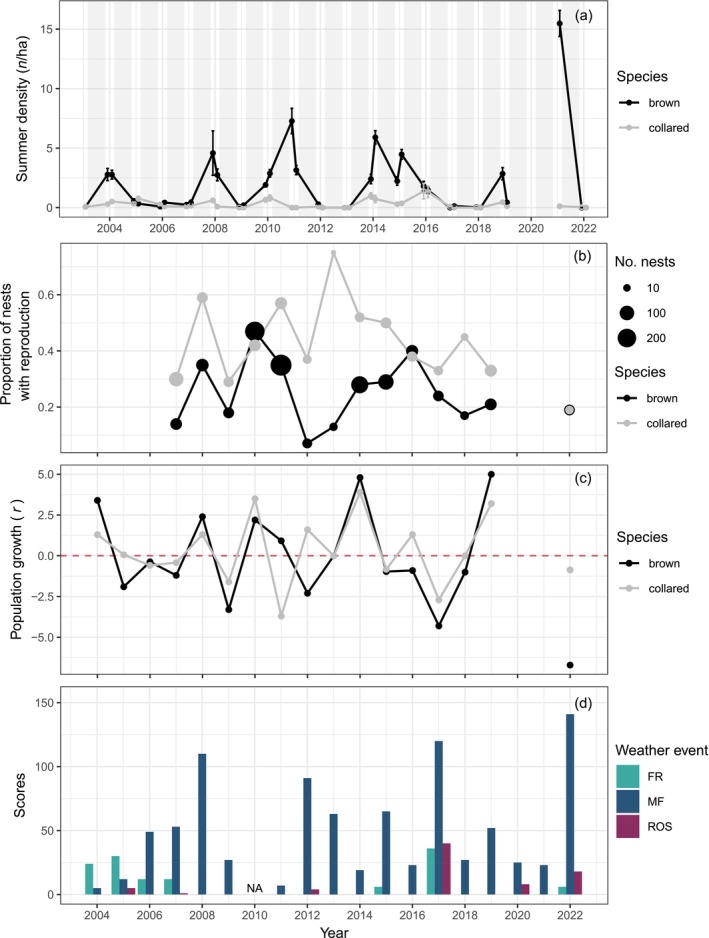
(a) Mean summer density in June and August from 2003 to 2022, (b) proportion of winter nests with reproduction from 2007 to 2019, and (c) mean winter population growth (*r*) from 2004 to 2022 in humid and mesic grids for brown and collared lemmings on Bylot Island. In (a) gray boxes represent winter, and in (c) red line represents a null winter population growth. (d) Scores of the different weather events occurring at the beginning of winter (from snow onset to the end of November) on Bylot Island over the period 2004–2022. No measurement is available for 2010 (NA). Freezing rain (FR) is the sum of hours with freezing rain events, melt‐freeze (MF) is the sum of positive temperature recorded every hour with humidity <95%, and rain‐on‐snow (ROS) is the sum of positive temperature recorded every hour with humidity >95% (see *Method* for details). In (a) error bars represent SE. In (b–d), each winter is referred to by the year when it ended. See Appendix S2 for additional variables.

During winters 2004–2022, we documented 6 years with rain‐on‐snow events of various intensities from the snow onset date to the end of November on Bylot Island (Figure 1). Melt‐freeze events were more common and occurred every year but with a highly variable intensity (scores between 4 and 140; Figure 1). Finally, we detected 7 years with freezing rain events near our study site (Figure 1). The strongest rain‐on‐snow events occurred exclusively during lemming crash phases (beginning or end of this phase), whereas melt‐freeze and freezing rain occurred during various phases of the lemming population cycle (Figure 1).

### Winter reproduction

**FIGURE 2:**
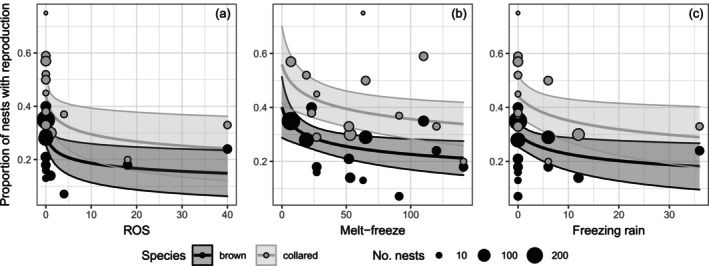
Logarithmic relationships between annual proportion of winter nests with reproduction and (a) rain‐on‐snow (ROS), (b) melt‐freeze, and (c) freezing rain scores at the beginning of winter in brown and collared lemmings on Bylot Island, 2007–2022. Values on the *x*‐axis were back‐transformed from a logarithmic scale. Overlapping data points were adjusted on the graph by adding or subtracting 0.1 to the original value. Size of dots is proportional to sample size. Filled‐in areas are 95% CI.

From 2007 to 2022, the proportion of winter nests with signs of reproduction was 1.7 times greater on average in collared compared to brown lemmings (Table 1, Figure 1). We found evidence for negative, nonlinear relationships between winter reproduction and the intensity of rain‐on‐snow, melt‐freeze, or freezing rain in both species (Figure 2, Table 1). On average, the proportion of winter nests with reproduction was reduced by 47% by rain‐on‐snow, 43% by melt‐freeze, and 37% by freezing rain events over the range of scores encountered for each of these variables on Bylot Island. We also found weak evidence for a slight positive, nonlinear relationship between winter reproduction and lemming density (Table 1, Appendix S2: Figure S1), but no evidence for an interaction with weather event variables. We did not find evidence for an influence of snow depth in November and snow onset date (Appendix S2: Figures S2 and S3) on winter reproduction (Appendix S2: Table S1). We also did not find support for interactive effects between snow variables and ermine abundance, as well as no influence of ermine alone on winter reproduction (Table 1, Appendix S2: Table S1). We detected the presence of some influential years (Cook's distance >0.15), including one year with particularly strong weather events (2017). However, excluding these years from our main models yielded the same trends and similar effect size (Appendix S2: Table S2).

**TABLE 1:** Coefficients of models examining the influence of weather events (rain‐on‐snow [ros], melt‐freeze [melt], and freezing rain [fr]), snow depth in November, and snow onset date on annual proportion of lemming winter nests with reproduction, with additive or interactive effects of lemming species, density in August of the previous year (density), and ermine abundance of the previous summer on Bylot Island, 2007–2022.

Model	Parameter	β	95% CI	φ	*k*	*R* ^2^
ros + density + species	**log(ros)**	**−0.23**	**[−0.38, −0.08]**	1.91	4	0.43
	**log(density)**	**0.09**	**[0.00, 0.18]**			
	**collared**	**0.76**	**[0.40, 1.12]**			
ros + ermine + species	**log(ros)**	**−0.26**	**[−0.42, −0.09]**	1.96	4	0.41
	ermine	0.20	[−0.02, 0.43]			
	**collared**	**0.64**	**[0.31, 0.97]**			
ros × density + species	**log(ros)**	**−0.23**	**[−0.39, −0.07]**	1.99	5	0.40
	**log(density)**	**0.10**	**[0.00, 0.20]**			
	**collared**	**0.77**	**[0.39, 1.15]**			
	log(ros) × log(density)	0.02	[−0.08, 0.11]			
fr + density + species	**log(fr)**	**−0.32**	**[−0.56, −0.09]**	2.04	4	0.39
	**log(density)**	**0.10**	**[0.00, 0.19]**			
	**collared**	**0.80**	**[0.42, 1.17]**			
ros × ermine + species	log(ros)	−0.33	[−0.71, 0.06]	2.04	5	0.39
	ermine	0.20	[−0.03, 0.43]			
	**collared**	**0.65**	**[0.31, 0.98]**			
	log(ros) × ermine	0.05	[−0.19, 0.28]			
fr × density + species	**log(fr)**	**−0.31**	**[−0.55, −0.07]**	2.12	5	0.37
	log(density)	0.04	[−0.19, 0.28]			
	**collared**	**0.82**	**[0.43, 1.21]**			
	log(fr) × log(density)	0.04	[−0.12, 0.20]			
ros + species	**log(ros)**	**−0.20**	**[−0.36, −0.04]**	2.12	3	0.36
	**collared**	**0.60**	**[0.26, 0.94]**			
melt + density + species	**log(melt)**	**−0.24**	**[−0.43, −0.04]**	2.16	4	0.35
	log(density)	0.06	[−0.04, 0.15]			
	**collared**	**0.73**	**[0.34, 1.12]**			

*Note*: The slope estimate (β), its 95% CI, the dispersion parameter (φ), the number of parameters (*k*), and the adjusted *R* ^2^ are presented. Models appear in decreasing order of *R* ^2^. Conclusive fixed effects are in bold. See Appendix S2: Table S1 for the exhaustive model list.

### Winter population growth

**FIGURE 3:**
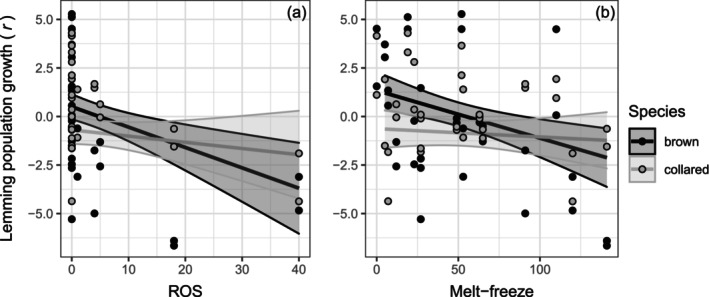
Linear relationships between winter population growth (*r*) of brown and collared lemmings and (a) rain‐on‐snow (ROS) and (b) melt‐freeze scores at the beginning of winter on Bylot Island, 2004–2022. Overlapping data points were adjusted on the graph by adding or subtracting 0.01 to the original value. Filled‐in areas are 95% CI.

Winter population growth (*r*) fluctuated from −6.7 to 5.3 for brown lemmings and from −4.4 to 4.3 for collared lemmings between 2004 and 2022 (Figure 1, Appendix S2: Figure S4). We found evidence for negative relationships between winter population growth and rain‐on‐snow or melt‐freeze events in brown lemmings but not in collared lemmings, as shown by the presence of an interaction between species and weather events (Table 2, Figure 3). On average, brown lemming population growth was reduced from 0.5 to −3.7 by rain‐on‐snow and from 1.2 to −2.1 by melt‐freeze over the range of scores encountered for each of these variables on Bylot Island. We found evidence of a negative, nonlinear relationship between winter population growth and lemming density (Appendix S2: Figure S1), but no evidence for an interaction with weather events (Table 2, Appendix S2: Table S3). We did not find support for a relationship between winter population growth and either snow depth in November or snow onset date (Appendix S2: Table S3). We found little support for a negative relationship between winter population growth and ermine abundance in the previous summer or for an interaction between ermine and weather event or snow variables (Appendix S2: Table S3, Figure S5). We also detected the presence of some influential years (Cook's distance >0.06), including 2017. However, excluding these years from our main models yielded similar trends and effect sizes, but reduced evidence when removing both years simultaneously, likely due to a loss of statistical power (Appendix S2: Table S4). Finally, in our main models, the random effect of year explained 54%–58% of the variance, which was expected for a cyclic species, while grids only explained 4%–5%.

**TABLE 2:** Coefficients of models examining the influence of weather events (rain‐on‐snow [ros], melt‐freeze [melt], and freezing rain [fr]), snow depth in November (depth), and snow onset date on winter population growth of lemmings with additive or interactive effects of lemming species, density in August of the previous year (density), and ermine abundance of the previous summer on Bylot Island, 2007–2022.

Model	Parameter	β	95% CI	*k*	*R* ^2^_*m*_	*R* ^2^_*c*_
ros × species + density	(Intercept)	−0.70	[−1.66, 0.26]	7	0.62	0.85
	**ros**	**−0.10**	**[−0.18, −0.01]**			
	**collared**	**−1.38**	**[−2.36, −0.41]**			
	**log(density)**	**−1.08**	**[−1.32, −0.83]**			
	**ros × collared**	**0.07**	**[0.01, 0.14]**			
melt × species + density	(Intercept)	0.13	[−1.18, 1.44]	7	0.60	0.85
	**melt**	**−0.02**	**[−0.05, 0.00]**			
	**collared**	**−2.04**	**[−3.41, −0.67]**			
	**log(density)**	**−1.04**	**[−1.29, −0.79]**			
	**melt × collared**	**0.02**	**[0.00, 0.04]**			
fr × species + density	(Intercept)	−0.91	[−2.16, 0.34]	7	0.56	0.84
	fr	−0.03	[−0.12, 0.06]			
	**collared**	**−1.57**	**[−2.62, −0.51]**			
	**log(density)**	**−1.16**	**[−1.43, −0.89]**			
	fr **×** collared	0.05	[−0.02, 0.11]			
ros + species + density	(Intercept)	−0.85	[−1.80, 0.11]	6	0.61	0.83
	ros	−0.06	[−0.11, −0.01]			
	**collared**	**−1.09**	**[−2.04, −0.14]**			
	**log(density)**	**−1.07**	**[−1.34, −0.81]**			
melt + species + density	(Intercept)	−0.44	[−1.46, 0.59]	6	0.59	0.83
	melt	−0.01	[−0.03, 0.00]			
	**collared**	**−1.11**	**[−2.08, −0.15]**			
	**log(density)**	**−1.09**	**[−1.35, −0.83]**			

*Note*: Grid and year were used as random factors. The slope estimate (β), its 95% CI, the number of parameters (*k*) and both *R* ^2^_*m*_ and *R* ^2^_*c*_ are presented. Models appear in decreasing order of *R* ^2^_*c*_. Conclusive fixed effects are in bold. See Appendix S2: Table S3 for the exhaustive model list.

### Demographic response to snow basal density

**FIGURE 4:**
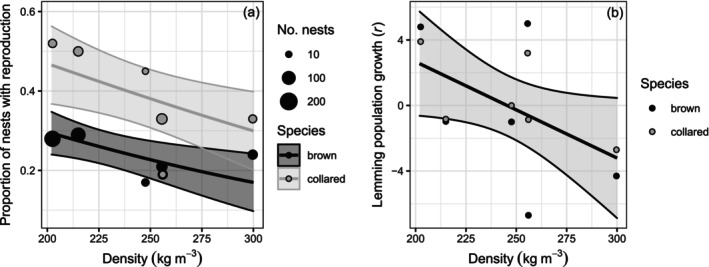
Linear relationships between annual proportion of winter nests with reproduction (a) or winter population growth (b) and snow basal density for brown and collared lemmings on Bylot Island, 2014–2022. In (a) size of dots is proportional to sample size. Filled‐in areas are 95% CI.

During winters 2014–2022, snow basal density varied between 200 and 300 kg m^−3^ (Appendix S2: Figure S6). We found weak evidence for a negative influence of snow basal density on the proportion of winter nests with reproduction (β = −0.01, CI = [−0.01, 0.00]; Figure 4a) and stronger evidence for a negative influence on winter population growth (β = −0.06, CI = [−0.10, −0.02]; Figure 4b).

## DISCUSSION

### Linking lemming winter demography to snow conditions

Our results support the hypothesis that snow conditions play a significant role in lemming demography during winter. More specifically, we found evidence for detrimental effects of early winter weather events that lead to snow hardening on lemming winter reproduction and population growth. For population growth, these effects were strong in brown lemmings, but no clear evidence was found for collared lemmings. Rain‐on‐snow events may not be as frequent or intense in the Canadian Arctic compared to regions exposed to oceanic currents (e.g., Northern Europe), but their occurrence still appears to reduce lemming population growth and intensity of reproduction during winter. Melt‐freeze events following the onset of the snowpack, which are more frequent than rain‐on‐snow events, also appear to influence lemming demography in winter. Finally, we also found evidence that freezing rain, which leads to the formation of thin ice layers above the snow, reduces lemming winter reproduction. These findings are consistent with those of Domine, Gauthier, et al. (2018), who also observed a decline in lemming populations during winter in the presence of a hard basal snow layer inferred through a snow physics model. However, whereas Domine, Gauthier, et al. (2018) identified rain‐on‐snow as the main source of snow hardening, our study also highlights the importance of melt‐freeze and freezing rain events in disturbing the snowpack and lemming demography.

Previous studies have reported collapses of small mammal populations following heavy rain‐on‐snow events in Arctic regions with milder and wetter climates than the Canadian High Arctic, such as on Svalbard (Stien et al., 2012) and in Norway (Ims et al., 2008). Extreme rain‐on‐snow events are of significant importance because they can encapsulate vegetation in an ice layer, depriving small mammals of an access to food and leading to catastrophic winter mortality (Ims et al., 2008; Kausrud et al., 2008). Larger herbivores such as reindeer (*Rangifer tarandus*) and muskoxen (*Ovibos moschatus*) also suffer from the formation of basal ice layers following severe rain‐on‐snow events (Rennert et al., 2009; Stien et al., 2012). In the Canadian Arctic, where the climate is particularly cold and dry, these weather events are less extreme than in other Nordic regions with milder and wetter climates. Moderate rain‐on‐snow events occur when a smaller amount of liquid water accumulates in the snow, which is more likely to create a hard melt‐freeze layer at the base of the snowpack rather than a solid ice layer, which has never been observed in such a region (Domine et al., 2016; Domine, Gauthier, et al., 2018). In the presence of a dense and hard basal snow layer, lemmings may still be able to access vegetation, but at an increased cost because the digging efficiency of lemmings decreases in hard snow despite an increase in their digging effort (Poirier et al., 2021). This could reduce the distance traveled within the snowpack, which may decrease the chance of finding adequate food sources or of encountering a mate. In addition, the increased energy spent digging may reduce the energy available for reproduction. These mechanisms could explain the reduction in the intensity of winter reproduction that we observed in the presence of weather events in early winter. Therefore, our results support the idea that mild‐to‐moderate weather events leading to a hardening of the basal snow layer early in the season could lead to reduced population growth or even a decline during winter caused by a reduction in reproduction intensity.

Other weather events not considered in our study, such as strong winds, can also lead to snow hardening (Domine et al., 2016). However, hardening of the snow basal layer by wind did not seem to impact lemming population growth (Domine, Gauthier, et al., 2018). Timing of formation of a hard snow layer can also have varying implications for lemmings. Hardening of upper snow layers after the snowpack is established can occur with strong winds or rain‐on‐snow events in warmer regions (Domine et al., 2016; Liston & Hiemstra, 2011). These resulting hard wind slab or melt‐freeze layers may actually benefit lemmings by hindering the ability of predators like foxes to hunt them through the snow (Bilodeau et al., 2013a). Further investigation is needed to explore the consequences of the timing of weather events leading to hard snow layers, which may differ among Arctic species. For instance, for larger herbivores spending all their time at the surface of the snowpack, we can anticipate negative effects of hard snow layers irrespective of when or where they form in the snowpack (Rennert et al., 2009).

Contrary to our initial hypothesis, both snow onset date and early winter snow depth had no effect on lemming winter demography. Gilg et al. (2009) suggested that an early snow onset would be favorable for lemmings by providing prompt access to a refuge against cold and predators, which are especially numerous in fall. However, an early snow onset followed by several freeze–thaw cycles could be detrimental for small mammals as it would favor the formation of a hard basal snow layer (Gauthier et al., 2024). For snow depth, it is possible that small‐scale spatial heterogeneity due to wind‐driven snow redistribution in early winter was not captured by our measurements taken at a single spot with a snow gauge. Therefore, the relationship between snow onset date or snow depth and lemming winter demography may be more complex than previously thought.

### Density dependence and winter predation

Independently of snow conditions, we found that the density of the lemming population had a negative impact on its winter population growth but not on its reproduction, which actually showed a positive trend in relation to density. This suggests that the effect observed on population growth is driven primarily by density‐dependent mortality during winter, as lemmings can apparently maintain a high reproductive activity at high density. Moreover, we found no interaction between lemming density and weather events in their effects on population growth or reproduction, suggesting that the impact of weather events is similar at different lemming densities.

Food resources per se are not thought to be limiting for lemmings in winter at our study site (Bilodeau et al., 2014; Gauthier et al., 2025; Legagneux et al., 2012), though access to it may be more difficult in the presence of extreme weather events (see above). In the High Arctic, predation is considered as the main cause of mortality in lemming populations (Fauteux et al., 2016; Fauteux & Gauthier, 2022; Gilg et al., 2003). The absence of an effect of ermine in our analysis may not be surprising since Bolduc et al. (2025) recently showed that this species alone cannot drive lemming population cycles at our site, although it does affect the length of cyclic phases. The cyclic dynamics of lemmings in the High Arctic seem to be primarily driven by a guild of diverse and complementary avian and mammalian predators (Bergeron et al., 2025; Bolduc et al., 2025; Gilg et al., 2003; Therrien et al., 2014). Nonetheless, our results suggest that the presence of ermines, the only predator that can hunt lemmings under the snow, does not exacerbate the negative effects of early winter weather events on their population growth.

### Interspecific differences

The reproductive rate of both lemming species decreased in a similar way in the presence of weather events leading to an increase in snow hardness. Nonetheless, we observed a higher reproductive rate in collared lemmings than in brown lemmings regardless of snow conditions, which is consistent with the known biology of these species and findings from previous work (Poirier et al., 2023). The winter growth rate of brown lemmings was more impacted by rain‐on‐snow and melt‐freeze events than that of collared lemmings. This substantiates previous findings that collared lemmings are less impaired when digging through hard snow than brown lemmings (Poirier et al., 2021) and display greater physiological adaptations to cold environments (Hansen, 1957; Zimova et al., 2018). Despite their higher reproductive rate, collared lemmings tended to exhibit lower population growth rates than brown lemmings during winter, which is surprising. This strongly suggests that collared lemmings experience a higher mortality rate than brown lemmings during winter, likely due to predation. Winter nests with reproductive activity have been associated with a higher predation risk by ermines (MacLean et al., 1974; Schmidt et al., 2021), which could lead to higher mortality in collared lemmings. Noise or odors owing to the presence of juveniles in a nest could attract predators, thereby increasing predation risks. Additionally, competition with brown lemmings could be another source of mortality in collared lemmings as the former are known to be more aggressive (Morris et al., 2000). We noticed instances of brown lemmings taking over collared lemming nests, and we observed at least one case of infanticide at our study site (M. Poirier, pers. obs).

### Limitations of the study

Despite our long‐term efforts, we recognize that 17 years of data is still somehow limited for a cyclic species. Indeed, all possible combinations of intensity of weather events and cyclic phases are not present in our dataset, especially for rain‐on‐snow events, and thus some uncertainty remains in our interpretation. For instance, years with the strongest rain‐on‐snow events, such as 2017 and 2022, coincided with the decline phase of the cycle. However, even after excluding these years from the analysis, we still found evidence for an impact of weather events on lemming winter demography. Furthermore, it is noteworthy that lemming crashes were more severe during these two years than in any other year, suggesting that these weather events may have played a role in the magnitude of the declines that we observed. However, we acknowledge that having more years with stronger rain‐on‐snow events during other phases of the population cycle would help better evaluate their overall effects on lemming demography.

We must also recognize that our reproduction index based on winter nests has some limitations. For instance, if the use of winter nests for reproduction differs between brown and collared lemmings, this could bias interspecific comparisons. However, considering the presence of permafrost and the near absence of rock piles at our study site, it is unlikely that a significant proportion of reproduction occurs outside of winter nests.

Finally, we also acknowledge a limitation of our study concerning the detection of rain‐on‐snow events. Because reliable precipitation data were not available at our study site, we had to use relative humidity in combination with air temperature to infer the presence of precipitation and its phase, which could be a source of error. For freezing rain data, we used a radius of 80 km from the center of our study site to account for limited temporal resolution, but such weather events can be very localized (Roberts & Stewart, 2008). Hence, we cannot guarantee that the identified freezing rain events occurred directly at our study site. However, for all three distinct types of weather events used in our study, we can reasonably assume that those occurring at the beginning of winter when the snow cover is thin will lead to a hardening of the basal layer (Berteaux et al., 2017; Colbeck, 1982; Domine et al., 2009). This assumption is also supported by the same relationships found between lemming demography and our direct measurements of density of the basal snow layer even if it is based on a small number of years. Snow models such as CROCUS could in theory have been useful in providing more details on snow properties at the beginning of winter (Vionnet et al., 2012), but these models are still poorly suited to simulate processes occurring in the Arctic snowpack (Domine et al., 2019).

## CONCLUSION

Despite years of research into the mechanisms underlying lemming cycles and growing evidence that predation is the primary driver at multiple sites (Bergeron et al., 2025; Fauteux et al., 2016; Gilg et al., 2003), uncertainties remain to fully understand these cycles. Our study provides key evidence on the influence of snow conditions on lemming population dynamics in the High Arctic mediated through an impact on their winter reproduction, a crucial demographic parameter in their periodic irruptions (Fauteux et al., 2015; Ims et al., 2011; Miller, 2001). More importantly, it highlights the growing concern regarding the influence of climate change on lemming populations (Gauthier et al., 2024; Gilg et al., 2009; Kausrud et al., 2008). Rain‐on‐snow, melt‐freeze, and freezing rain events are expected to increase in the Arctic (Groisman et al., 2016; Hansen et al., 2014; Peeters et al., 2019), which should result in an increasing frequency of hard snow layers. These conditions should reduce the intensity of winter reproduction in lemmings and could even lead to mortality from starvation if basal ice forms during extreme weather events. Consequently, this could considerably dampen the periodic peaks during cyclic population fluctuations of lemmings (Hörnfeldt et al., 2005; Ims & Fuglei, 2005; Kausrud et al., 2008), with strong negative consequences for numerous predators such as snowy owls (*Bubo scandiacus*) that depend upon them for their reproduction (Schmidt et al., 2012; Therrien et al., 2014). To further enhance our understanding of the role played by these parameters in lemming population fluctuations, we encourage researchers to incorporate snow variables or weather event indices in their modeling (Bergeron et al., 2025).

In northern regions, snow covers the ground for more than half of the year, significantly affecting the habitat of species that live above or below snow. Changes in snow conditions could influence not only species movements and energy expenditure but also feeding activity and interspecific interactions (Marchand, 2013). For instance, predator–prey interactions have been shown to be influenced by snow conditions, and alterations in snow hardness or persistence could severely modify these dynamics (Peers et al., 2020; Sullender et al., 2023). There are numerous ways in which snow conditions could influence population dynamics, yet little effort has been devoted to the field of winter ecology so far (Reinking et al., 2022; Studd et al., 2021). Our findings highlight the significant role of snow and climatic factors in population dynamics and also expose a need for further research into these complex snow–wildlife interactions. New technologies, such as subnivean camera traps (Kalhor et al., 2024; Mölle et al., 2021), offer promising avenues to increase our understanding of this critical phase of the life cycle of boreal species.

## CONFLICT OF INTEREST STATEMENT

The authors declare no conflicts of interest.

## Supporting information


Appendix S1.

Appendix S2.

## Data Availability

Data are available in the NordicanaD repository as follows: lemming data (Gauthier, 2020), https://doi.org/10.5885/45400AW-9891BD76704C4CE2; weather station data (Centre d'études nordiques, 2024), https://doi.org/10.5885/45039SL-EE76C1BDAADC4890; snow and weather station data (Domine et al., 2021a, 2021b), https://doi.org/10.5885/45693CE-02685A5200DD4C38.
