# Supplementary material for: Demography of lemmings in response to changing snow conditions in the High Arctic
Source: Ecology. 2025 Sep 23;106(9):e70216. doi: 10.1002/ecy.70216 (PMC12457247; doi:10.1002/ecy.70216)
Supplement: Supplementary file 1 — Appendix S1. [file ECY-106-e70216-s001.pdf]

Appendix S1

Demography of lemmings in response to changing snow conditions in the High Arctic

Mathilde Poirier, Gilles Gauthier, Florent Dominé, Dominique Fauteux

Ecology

In this study, the snow onset date corresponds to the first date of the season when snow covered more than 80 % of our study area, without returning below 50 % of coverage. Partial melting is part of the processes we wished to include in this study.

We used MODIS images (Moderate Resolution Imaging Spectroradiometer; MOD10A1 snow product collection 6 extracted from https://modis.gsfc.nasa.gov/) between 2003 and 2019 as a first clue of the timing of snow onset. The satellite provides one image daily and analysis of these data over our 51 km^2 study area allows detection of the presence of snow on the ground. We obtained snow cover information at the pixel level (500 m) with the normalized-difference snow index (NDSI) (see collection 6 User Guise of Riggs and Hall (2015) for more information). NDSI pixels were transformed into binary classes using a threshold of 0.4 to calculate the daily snow cover fraction (i.e., NDSI $\geq$ 0.4 classified as snow covered (1) and NDSI $<$ 0.4 classified as not snow covered (0)). The threshold of 0.4 is a standard value widely used to distinguish snow from other bright material like clouds, soils or rocks (Dozier 1989). However, estimating a snow onset date with this data does not always give an accurate result as there are often gaps between two usable images due to the presence of clouds (gaps ranging from 1 to 18 days).

We used other cues to refine the snow onset dates derived from MODIS images. Since 1993, an automated station records hourly weather data at our study site. We used relative air humidity at 2 m, air temperature at 2 m, wind speed at 3 m and snow depth recorded hourly at the BYLCAMP station (CEN 2022) from 2000 to 2021 to help us narrow down our estimate of the snow onset date. In winter 2009-2010, no weather data was recorded due to a malfunction of the weather station and between 2013 and 2016, the same applies to the snow depth gauge. In those years, we used snow depth recorded from another weather station located about 1.7 km away from the main one (TUNDRA; Domine 2021).

In years with uncertainties in the data (2005, 2007, 2011) or with malfunction of the weather station (2009), we also used weather data recorded in Mittimatalik (Pond Inlet), about 90 km from our main study area as a complement (extracted from the Environment and Climate Change Canada Historical Climate Data web site https://climate.weather.gc.ca/index_e.html). Precipitation and other weather observations recorded in Mittimatalik were compared to weather data recorded on

Bylot Island to see the concordance between the two datasets and add more certainty to the detection of snow precipitation events.

From 2016 to 2021, an automated camera deployed at the study site took one photo daily of the surrounding landscape, which allowed us to get a precise snow onset date for these years. Between 2016 and 2019, we compared snow onset dates estimated with our method (based on MODIS images and local weather data) to those obtained with the automated camera. Snow onset dates were the same, which confirmed the reliability of our method.

In summary, we mixed several environmental cues to get the most accurate estimate of the snow onset date each year. Since this date influences other derived weather variables used in this study (melt-freeze, rain-on-snow and freezing rain), it was important to have the most accurate date possible. We provide below the estimated annual snow onset date along with the criteria used (Table S1), and the graphs of weather and MODIS data used to make these inferences each year (Figs S1 to S22).

Table S1. Yearly estimates of snow onset date (onset date). A description of the methods used to estimate the date (criteria used), as well as the corresponding figures of weather data (sources) are shown in the table.

Winter	Onset date	Criteria used	Source
2003-2004	October 6	- Oct 4: MODIS indicated a 10% snow cover.<br>- Oct 6: MODIS indicated a 100% snow cover; snow onset occurred between these dates.<br>- Oct 6: there is a humidity peak, air temperature is <0°C and the snow gauge detected a snow accumulation.	Fig S1
2004-2005	October 1	- Sept 29: MODIS indicated a 25% snow cover.<br>- Oct 9: MODIS indicated a 100% snow cover; snow onset occurred between these dates.<br>- Oct 1: there is a humidity peak, air temperature is <0°C and the snow gauge detected a snow accumulation.	Fig S2
2005-2006	September 26	- Sept 21: MODIS indicated a 50% snow cover.<br>- Oct 6: MODIS indicated a 100% snow cover; snow onset occurred between these dates.<br>- Sept 25-26: there is a humidity peak and air temperature is <0°C overnight.<br>- Sept 25-29: the snow gauge detected a gradual accumulation with some noise despite low air humidity values.<br>- Sept 27-28: a peak in wind speed occurred, suggesting that snow accumulated prior to this date was blown away.<br>- Sept 26: snow precipitation was recorded in Pond Inlet (no data available on Sept 25).	Figs S3, S4 & S5
2006-2007	September 30	- Sept 28: MODIS indicated a 0% snow cover.<br>- Oct 6: MODIS indicated a 100% snow cover; snow onset occurred between these dates.<br>- Sept 30-Oct 1: there is a humidity peak, air temperature is <0°C and the snow gauge detected a snow accumulation.	Fig S6
2007-2008	September 13	- Sept 11: MODIS indicated a 18% snow cover.<br>- Oct 14: MODIS indicated a 92% snow cover; snow onset occurred between these dates.<br>- Sept 13: there is a humidity peak, air temperature is <0°C and the snow gauge detected a snow accumulation with some noise.<br>- Sept 13: snow precipitation was recorded in Pond Inlet.	Figs S7, S8

2008-2009	September 15	- Sept 11: MODIS indicated a 0% snow cover.<br>- Sept 15: MODIS indicated a 100% snow cover; snow onset occurred between these dates.<br>- Sept 15: there is a humidity peak, air temperature is <0°C and the snow gauge detected a snow accumulation despite the noise.	Fig S9
2009-2010	September 21	- Sept 20: MODIS indicated a 0% snow cover.<br>- Sept 22: MODIS indicated a 95% snow cover; snow onset occurred between these dates.<br>- Sept 21: snow precipitation was recorded in Pond Inlet.<br>- No weather data was recorded on Bylot Island that year.	Fig S10
2010-2011	September 30	- Sept 29: MODIS indicated a 10% snow cover.<br>- Oct 1: MODIS indicated a 90% snow cover; snow onset occurred between these dates.<br>- Sept 30: there is a humidity peak (above 90%), air temperature is <0°C and the snow gauge detected a snow accumulation.	Fig S11
2011-2012	September 15	- Sept 11: MODIS indicated a 0% snow cover.<br>- Sept 22: MODIS indicated a 100% snow cover; snow onset occurred between these dates.<br>- Sept 15: there is a humidity peak, air temperature is <0°C and the snow gauge detected a snow accumulation.	Fig S12
2012-2013	September 27	- Sept 25: MODIS indicated a 9% snow cover.<br>- Sept 29: MODIS indicated a 100% snow cover; snow onset occurred between these dates.<br>- Sept 27: there is a humidity peak, air temperature is <0°C and the snow gauge detected a snow accumulation.	Fig S13
2013-2014	October 12	- Oct 10: MODIS indicated a 19% snow cover.<br>- Oct 14: MODIS indicated a 100% snow cover; snow onset occurred between these dates.<br>- Oct 12: there is a humidity peak, air temperature is <0°C and the snow gauge detected a snow accumulation.	Fig S14
2014-2015	September 12	- Sept 9: MODIS indicated a 0% snow cover.<br>- Sept 23: MODIS indicated a 100% snow cover; snow onset occurred between these dates.<br>- Sept 12: there is a humidity peak, air temperature is <0°C and the snow gauge detected a snow accumulation.	Fig S15

		- Sept 14-16: there was probably a snow melt episode between these dates, but the melt was only partial according to snow gauge.	
2015-2016	September 20	- Sept 17: MODIS indicated a 20% snow cover.<br>- Sept 21: MODIS indicated a 100% snow cover; snow onset occurred between these dates.<br>- Sept 18-19: there are humidity peaks, but the temperature remained mainly >0°C, suggesting liquid precipitation.<br>- Sept 20: there is a humidity peak and air temperature is <0°C overnight, suggesting snow precipitation.	Fig S16
2016-2017	September 9	- Sept 6: MODIS indicated a 11% snow cover.<br>- Sept 10: MODIS indicated a 100% snow cover; snow onset occurred between these dates.<br>- Sept 9: there is a humidity peak, air temperature is <0°C and the snow gauge detected a snow accumulation.<br>- Sept 9: photos from the automated camera detected a snow accumulation.<br>- Sept 18-20: there are humidity peaks with temperature >0°C, suggesting liquid precipitation.<br>- Sept 19-20: photos from the automated camera detected a partial snow melting.<br>- Sept 27-29: photos from the automated camera detected a rain-on-snow.	Fig S17
2017-2018	September 8	- Sept 5: MODIS indicated a 0% snow cover.<br>- Sept 8: MODIS indicated a 100% snow cover; snow onset occurred between these dates.<br>- Sept 8: there is a humidity peak, air temperature is <0°C and the snow gauge detected a snow accumulation.<br>- Sept 8: photos from the automated camera detected a snow accumulation.<br>- Sept 10-11: photos from the automated camera detected a partial snow melting.	Fig S18
2018-2019	September 16	- Sept 7: MODIS indicated a 0% snow cover.<br>- Sept 25: MODIS indicated a 100% snow cover; snow onset occurred between these dates.<br>- Sept 16: there is a humidity peak, air temperature is <0°C and the snow gauge detected a snow accumulation.	Fig S19

		- Sept 16: photos from the automated camera detected a snow accumulation.<br>- Sept 17-19: the snow gauge detected a decrease in snow depth, mostly due to strong wind speed or to a partial melting.<br>- Sept 18-19: photos from the automated camera detected a partial snow melting.	
2019-2020	September 30	- Sept 21: MODIS indicated a 0% snow cover.<br>- Oct 6: MODIS indicated a 100% snow cover; snow onset occurred between these dates.<br>- Sept 30: there is a humidity peak, air temperature is <0°C and the snow gauge detected a snow accumulation.<br>- Sept 30: photos from the automated camera detected a snow accumulation.	Fig S20
2020-2021	September 23	- MODIS analysis is not available for that year.<br>- Sept 23: photos from the automated camera detected a snow accumulation.<br>- Sept 23: there is a humidity peak, air temperature is <0°C and the snow gauge detected a small snow accumulation.	Fig S21
2021-2022	October 8	- MODIS analysis is not available for that year.<br>- Sept 30: there is a humidity peak, air temperature is <0°C and the snow gauge detected a snow accumulation.<br>- Sept 30-Oct 4: air temperature is >0°C and there are humidity peaks, suggesting melting and rain-on-snow episodes.<br>- Sept 30-Oct 4: photos from the automated camera detected a significant melting of the snow cover (<50% remaining).<br>- Oct 8: there is a humidity peak, air temperature is <0°C and the snow gauge detected a small snow accumulation.<br>- Oct 8: photos from the automated camera detected a snow accumulation.	Fig S22

Winter 2003-2004

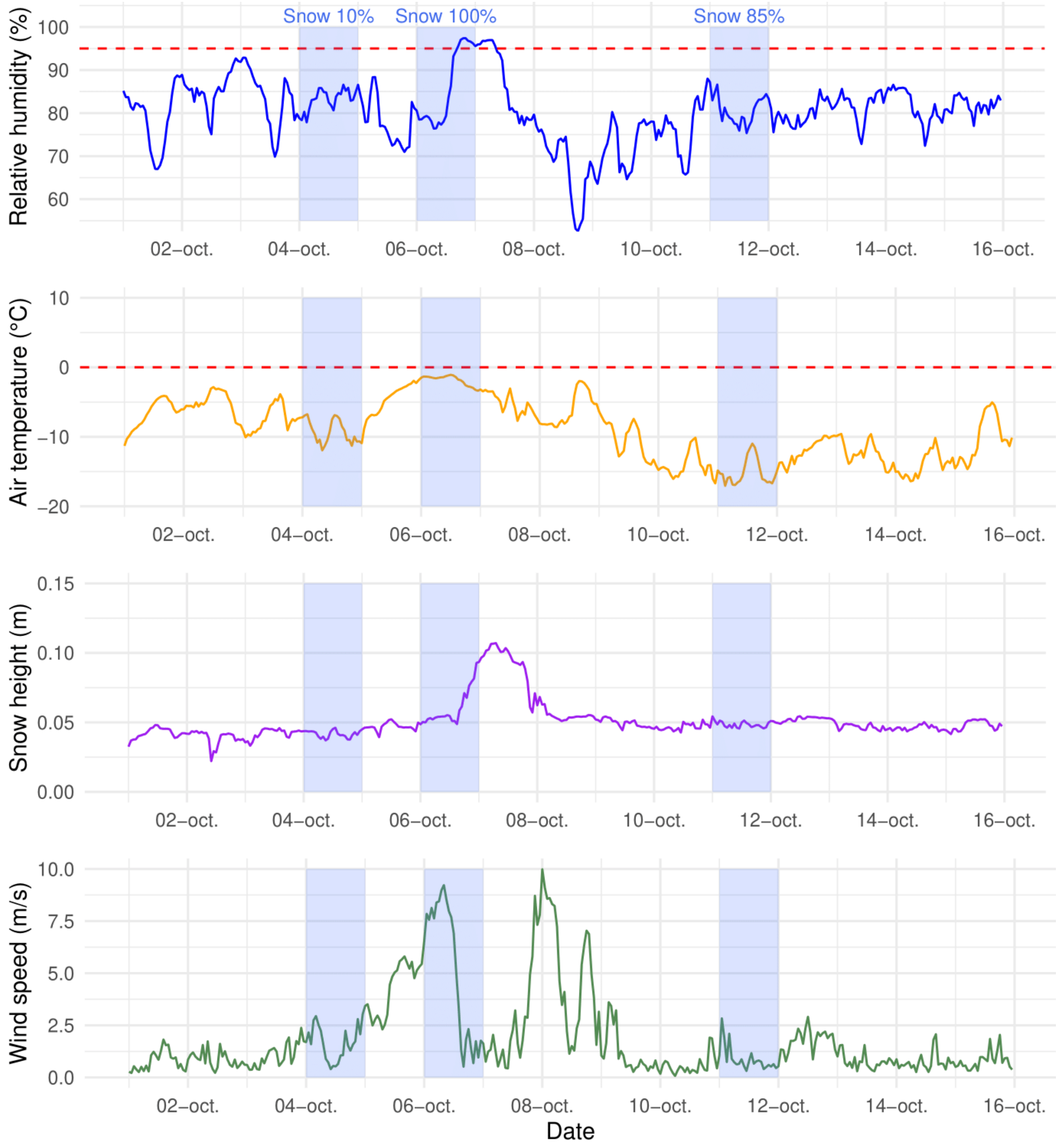

Figure S1. Weather conditions recorded during the period of snow onset in fall 2003. The blue boxes represent days with available MODIS images, and the percentage (%) of the study area covered by snow is indicated above the figure. The red dashed line on the relative humidity chart indicates the threshold (95%) above which we estimate a high risk of precipitation and on the air temperature chart it indicated 0°C.

Winter 2004-2005

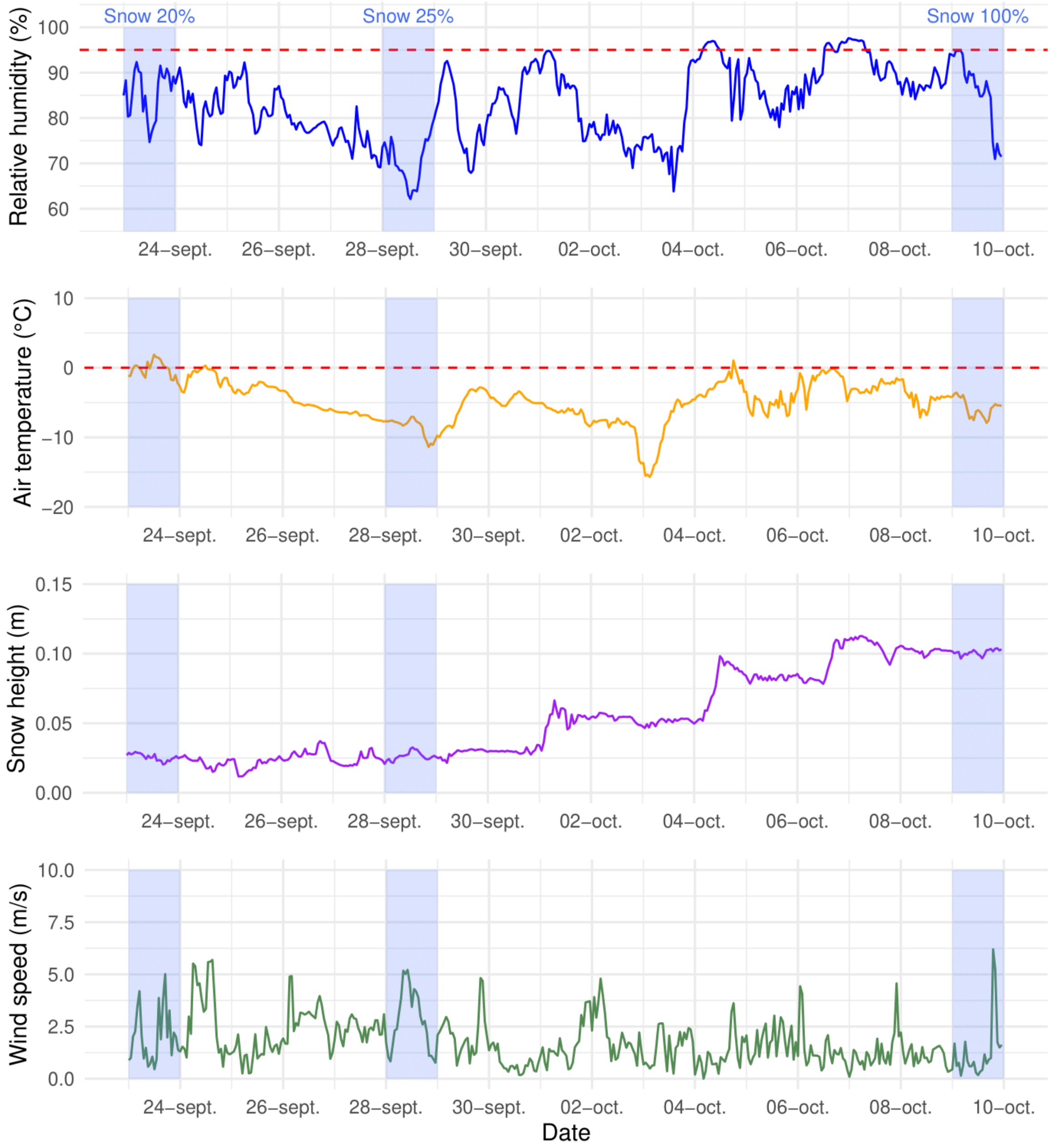

Figure S2. Weather conditions recorded during the period of snow onset in fall 2004. The blue boxes represent days with available MODIS images, and the percentage (%) of the study area covered by snow is indicated above the figure. The red dashed line on the relative humidity chart indicates the threshold (95%) above which we estimate a high risk of precipitation and on the air temperature chart it indicated 0°C.

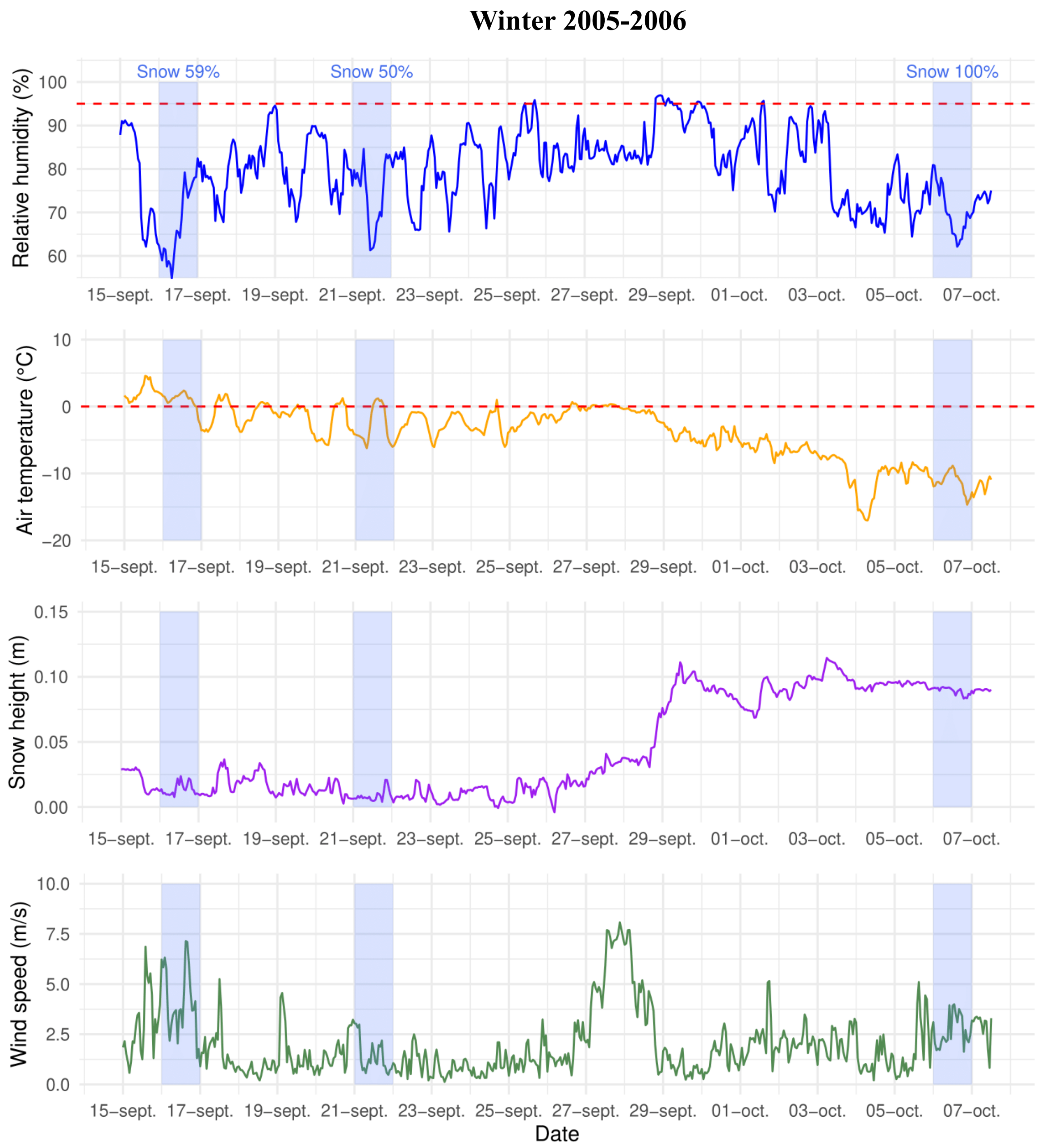

Figure S3. Weather conditions recorded during the period of snow onset in fall 2005. The blue boxes represent days with available MODIS images, and the percentage (%) of the study area covered by snow is indicated above the figure. The red dashed line on the relative humidity chart indicates the threshold (95%) above which we estimate a high risk of precipitation and on the air temperature chart it indicated 0°C.

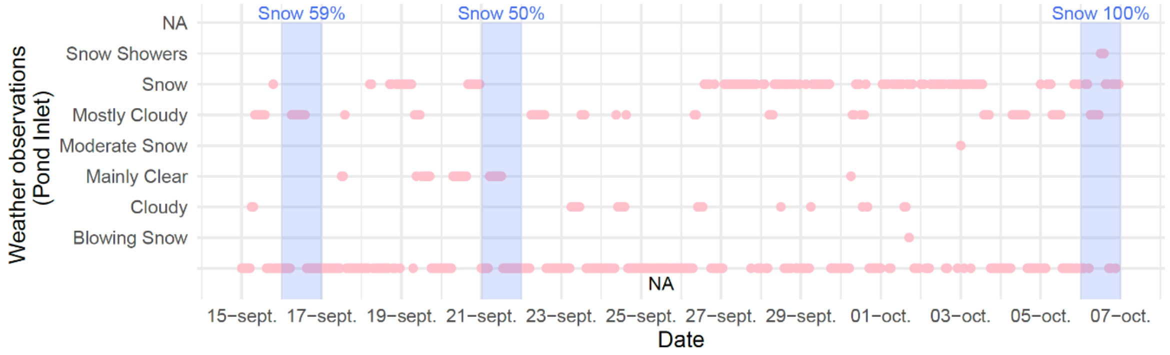

Figure S4. Weather observations from Pond Inlet in the period of snow onset in fall 2005. The blue boxes represent days with available MODIS images, and the percentage (%) of the study area covered by snow is indicated above the figure.

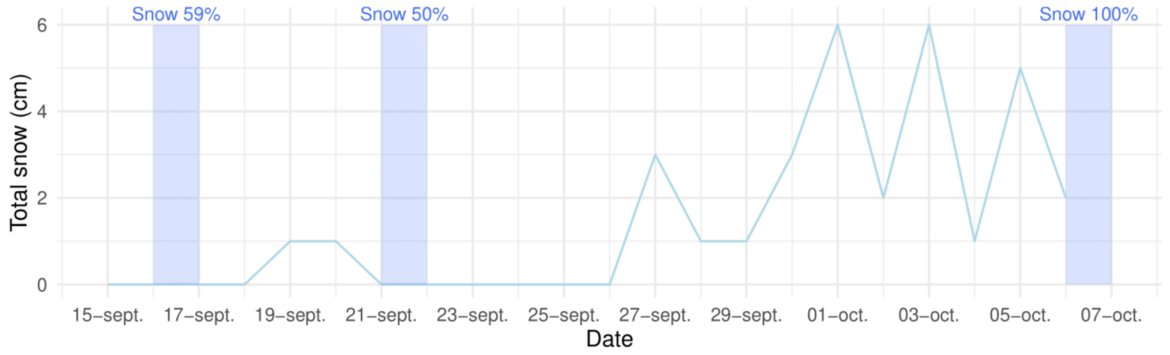

Figure S5. Total solid precipitation recorded in Pond Inlet in the period of snow onset in fall 2005. The blue boxes represent days with available MODIS images, and the percentage (%) of the study area covered by snow is indicated above the figure.

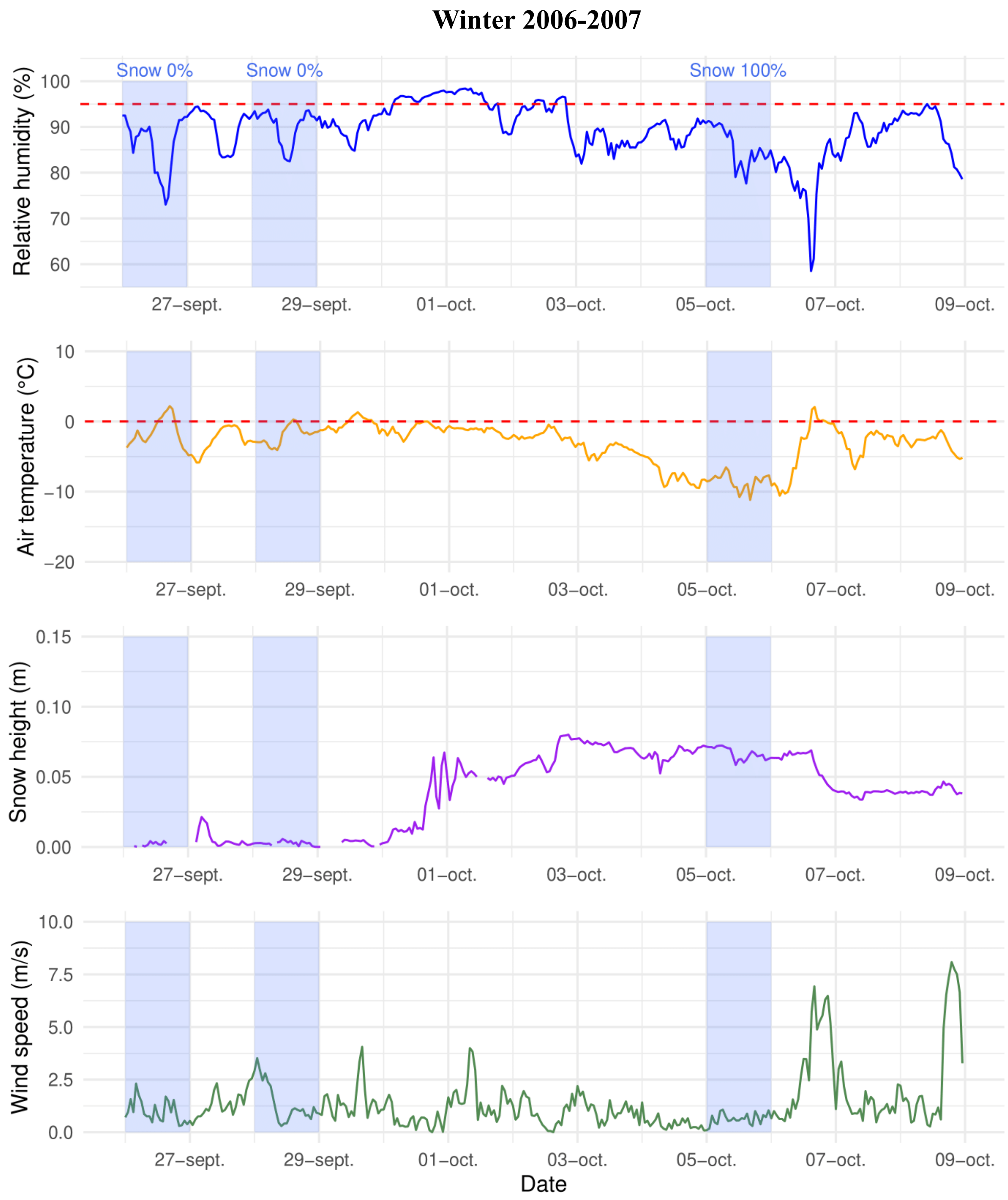

Figure S6. Weather conditions recorded during the period of snow onset in fall 2006. The blue boxes represent days with available MODIS images, and the percentage (%) of the study area covered by snow is indicated above the figure. The red dashed line on the relative humidity chart indicates the threshold (95%) above which we estimate a high risk of precipitation and on the air temperature chart it indicated 0°C.

Winter 2007-2008

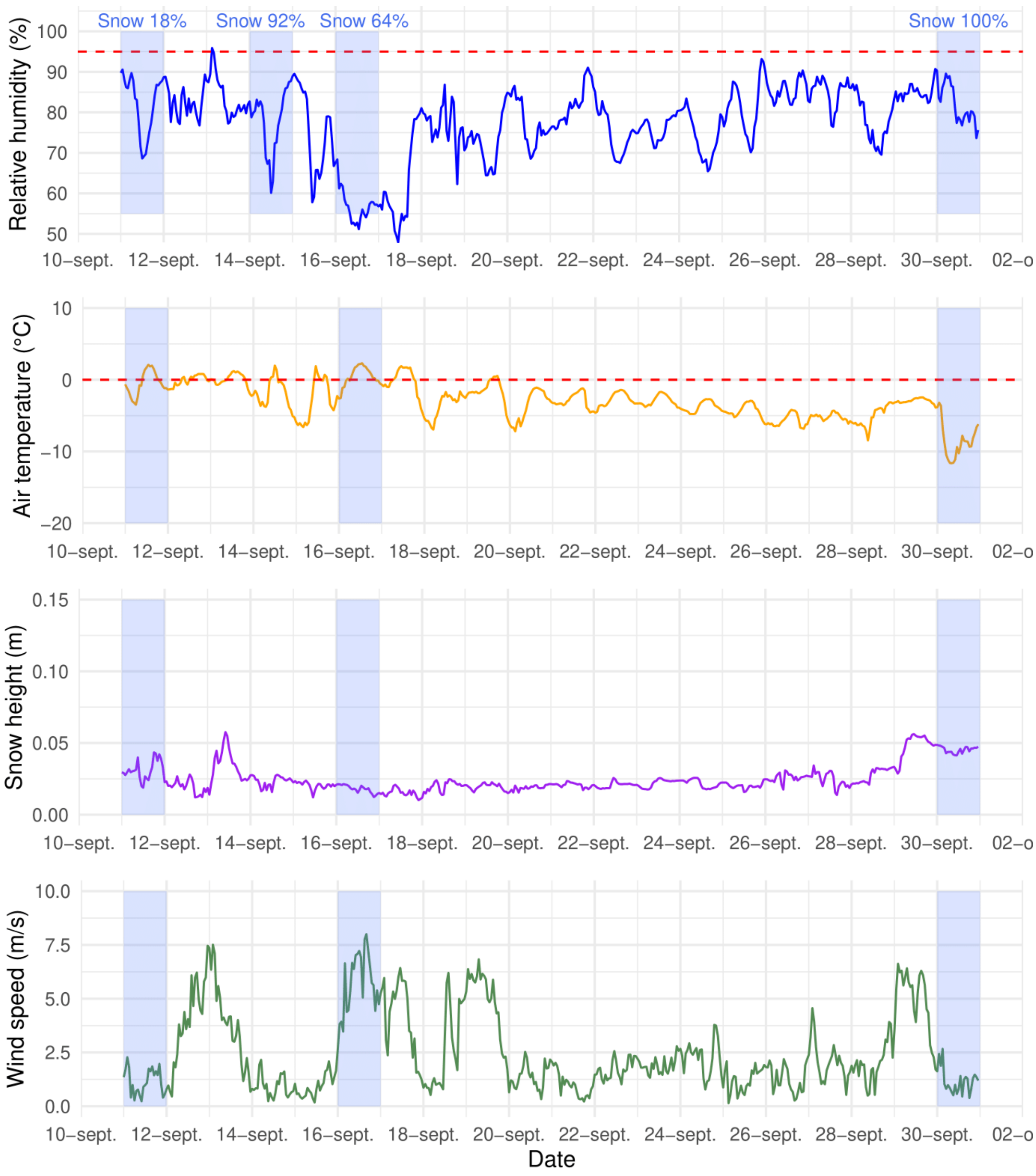

Figure S7. Weather conditions recorded during the period of snow onset in fall 2007. The blue boxes represent days with available MODIS images, and the percentage (%) of the study area covered by snow is indicated above the figure. The red dashed line on the relative humidity chart indicates the threshold (95%) above which we estimate a high risk of precipitation and on the air temperature chart it indicated 0°C.

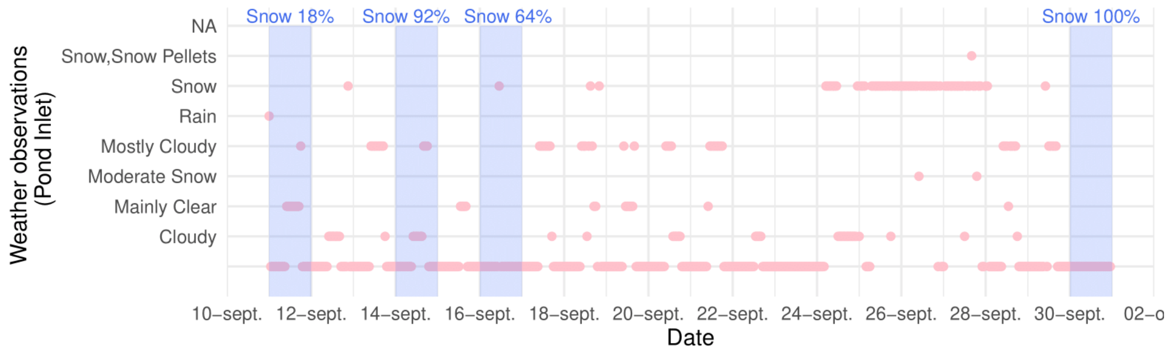

Figure S8. Weather observations from Pond Inlet in the period of snow onset in fall 2007. The blue boxes represent days with available MODIS images, and the percentage (%) of the study area covered by snow is indicated above the figure.

Winter 2008-2009

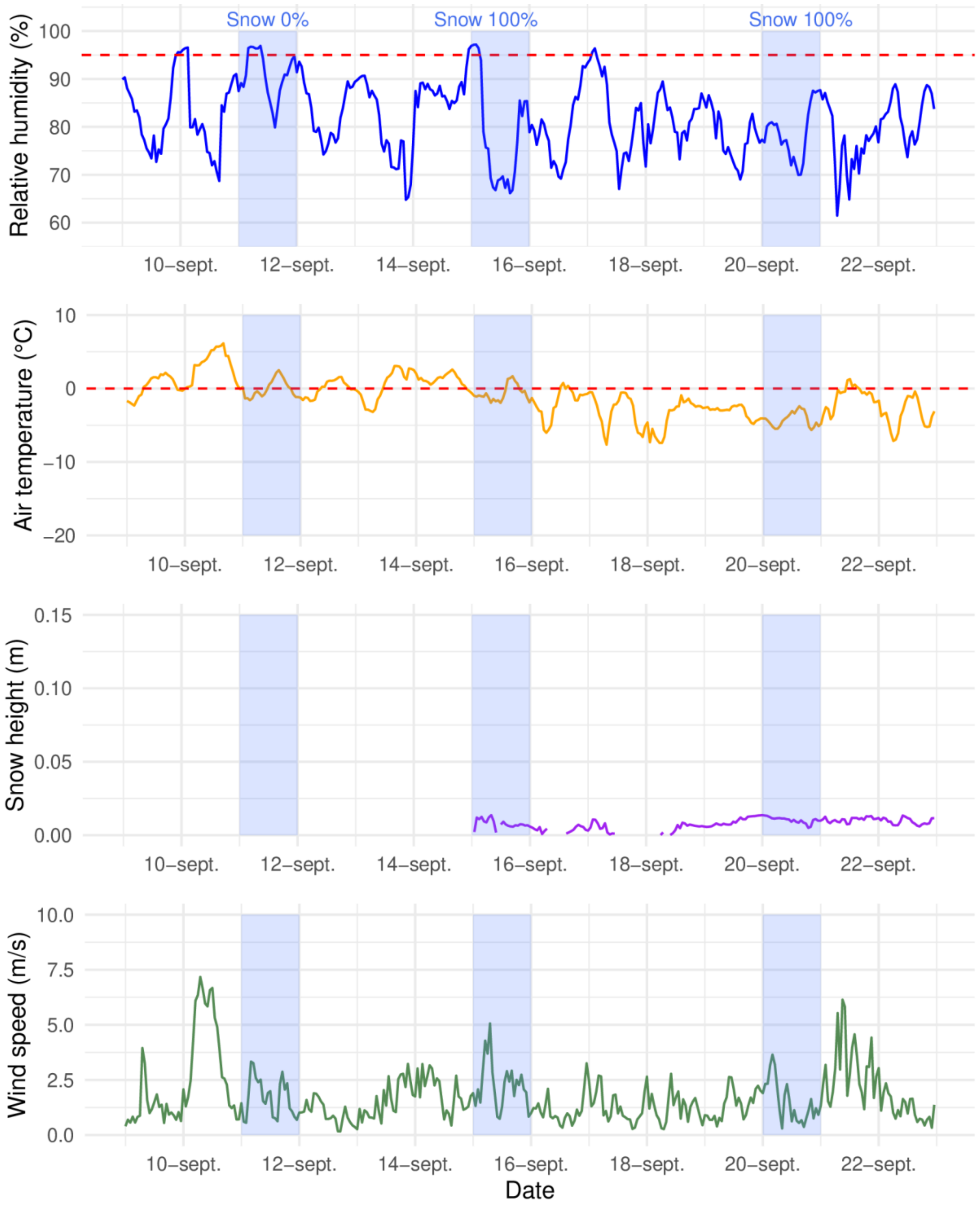

Figure S9. Weather conditions recorded during the period of snow onset in fall 2008. The blue boxes represent days with available MODIS images, and the percentage (%) of the study area covered by snow is indicated above the figure. The red dashed line on the relative humidity chart indicates the threshold (95%) above which we estimate a high risk of precipitation and on the air temperature chart it indicated 0°C.

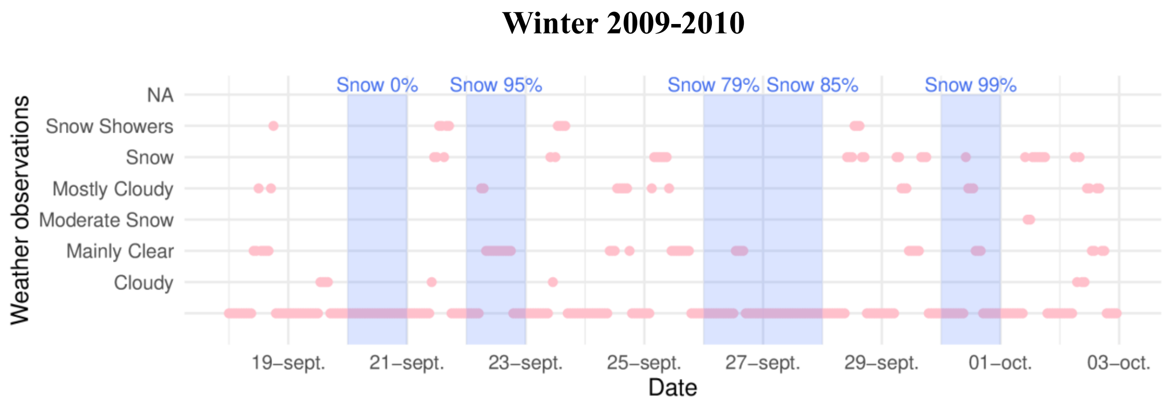

Figure S10. Weather observations from Pond Inlet in the period of snow onset in fall 2009. The blue boxes represent days with available MODIS images, and the percentage (%) of the study area covered by snow is indicated above the figure.

Winter 2010-2011

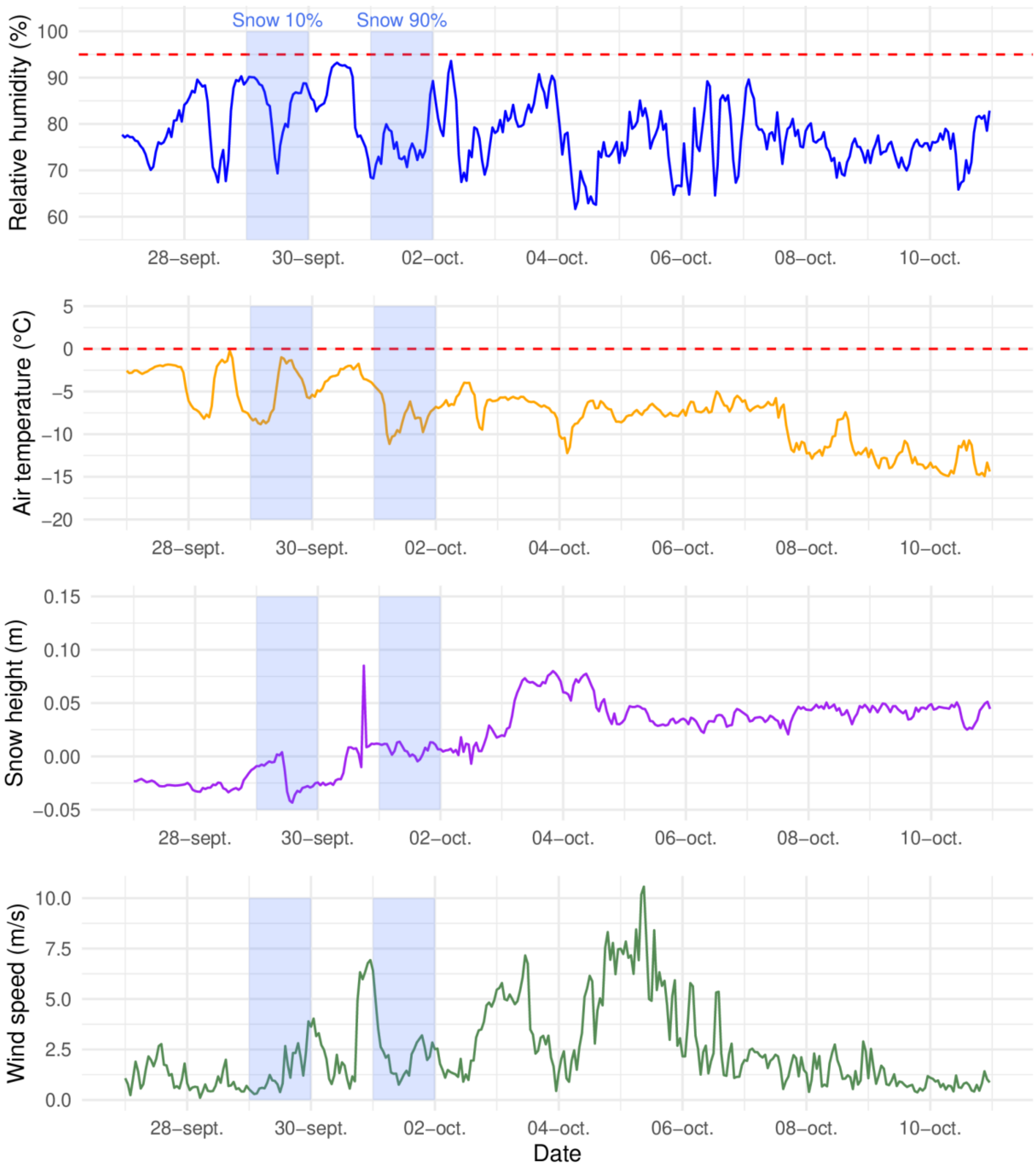

Figure S11. Weather conditions recorded during the period of snow onset in fall 2008. The blue boxes represent days with available MODIS images, and the percentage (%) of the study area covered by snow is indicated above the figure. The red dashed line on the relative humidity chart indicates the threshold (95%) above which we estimate a high risk of precipitation and on the air temperature chart it indicated 0°C.

Winter 2011-2012

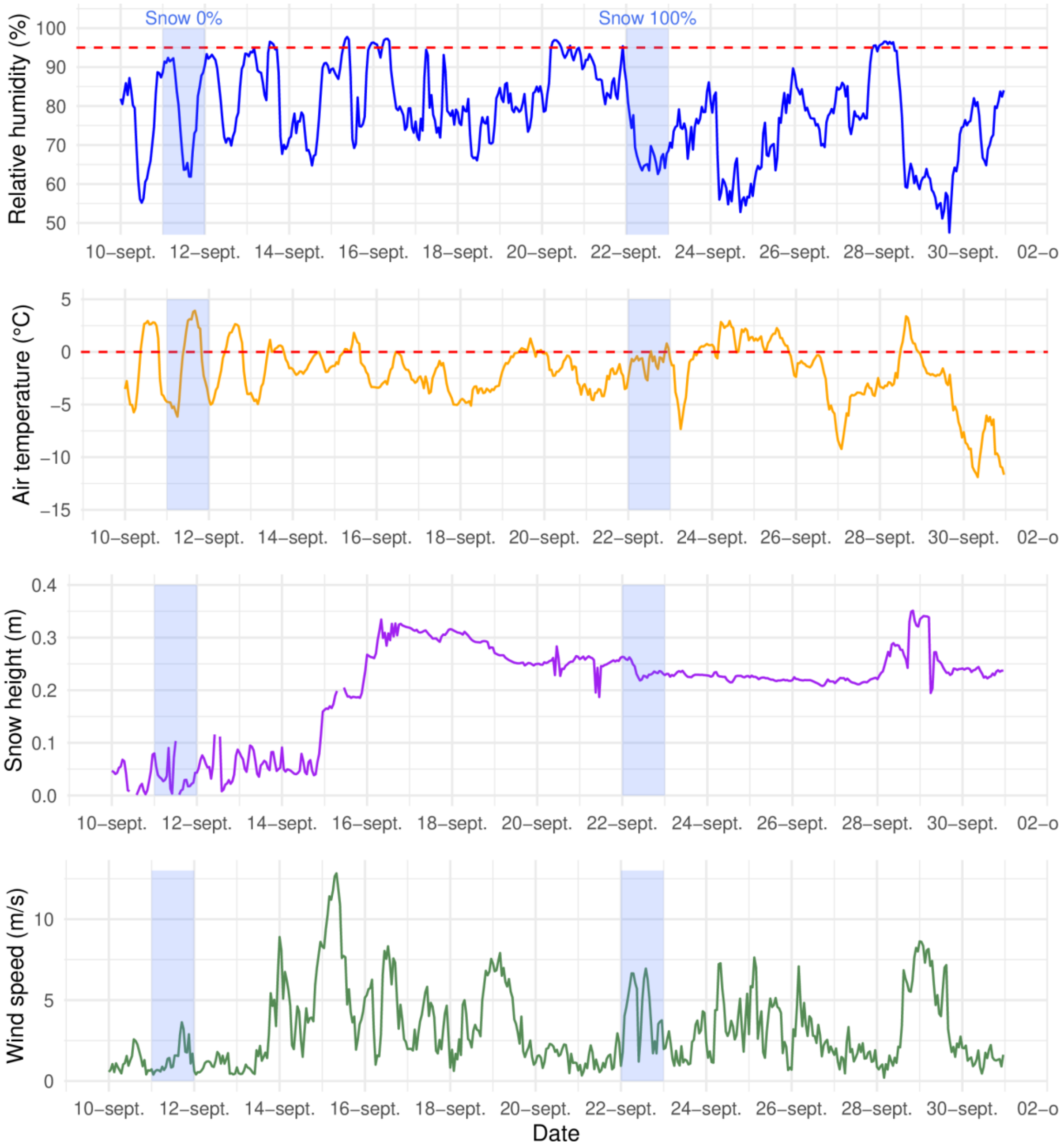

Figure S12. Weather conditions recorded during the period of snow onset in fall 2011. The blue boxes represent days with available MODIS images, and the percentage (%) of the study area covered by snow is indicated above the figure. The red dashed line on the relative humidity chart indicates the threshold (95%) above which we estimate a high risk of precipitation and on the air temperature chart it indicated 0°C.

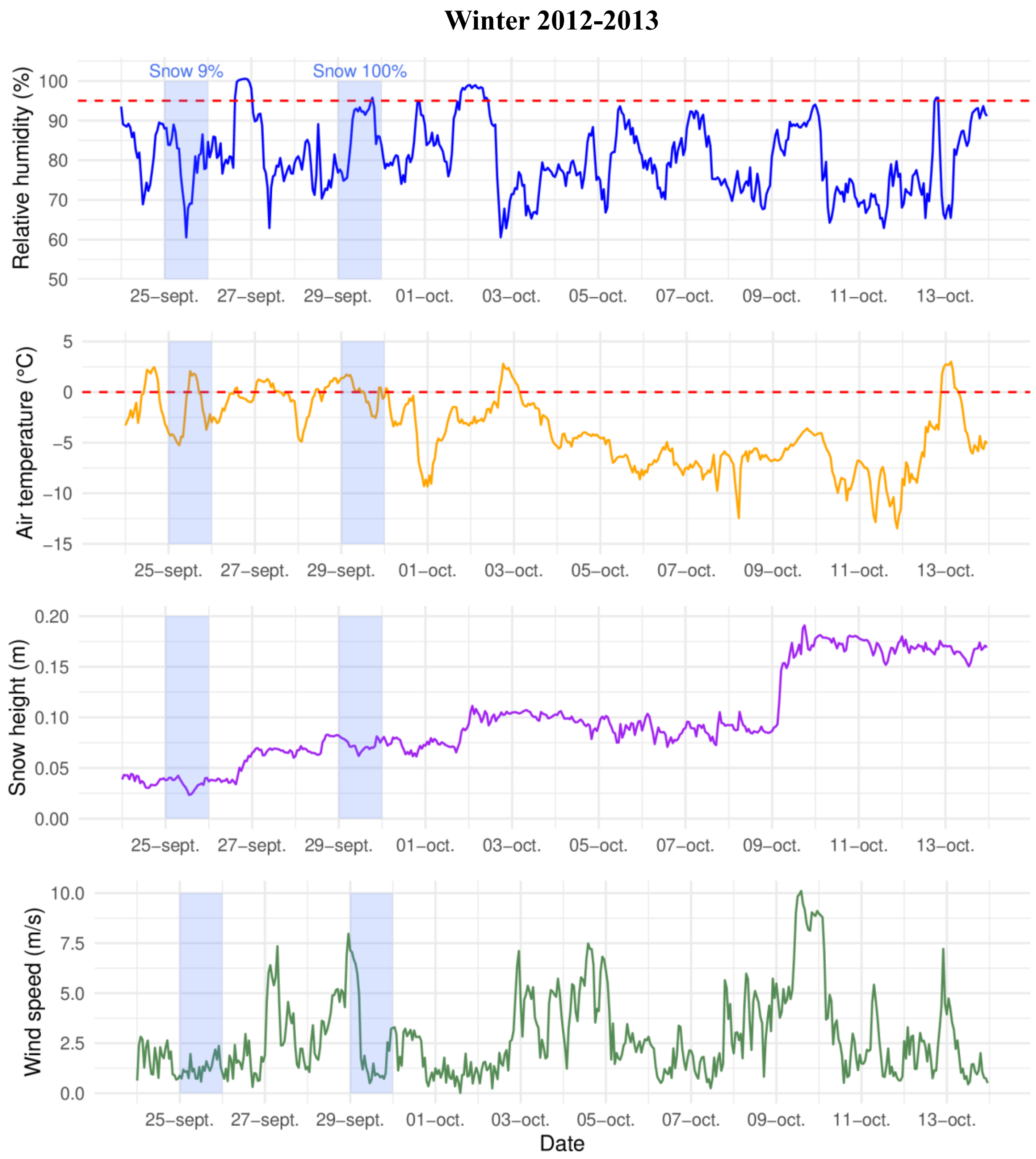

Figure S13. Weather conditions recorded during the period of snow onset in fall 2012. The blue boxes represent days with available MODIS images, and the percentage (%) of the study area covered by snow is indicated above the figure. The red dashed line on the relative humidity chart indicates the threshold (95%) above which we estimate a high risk of precipitation and on the air temperature chart it indicated 0°C.

Winter 2013-2014

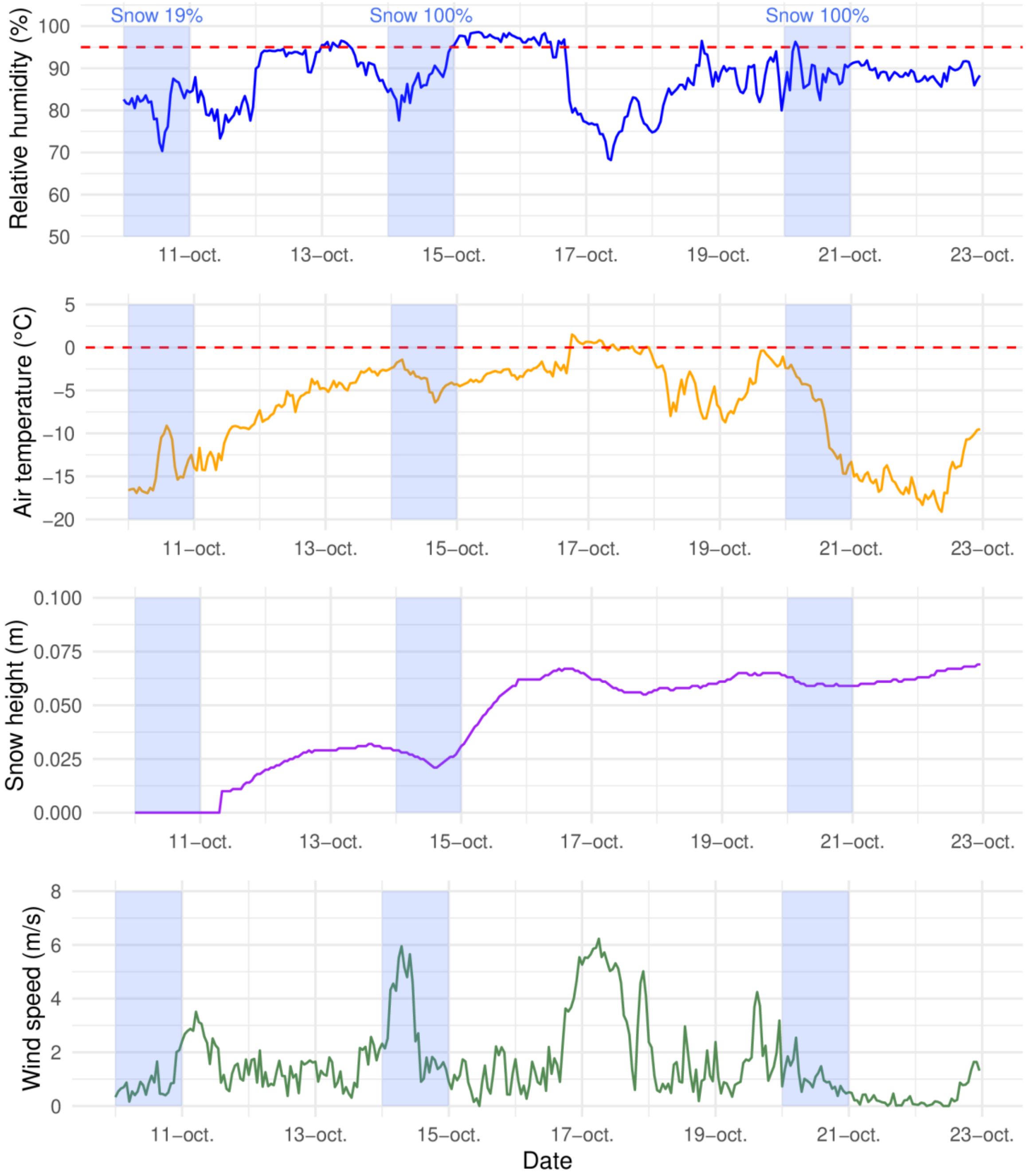

Figure S14. Weather conditions recorded during the period of snow onset in fall 2013. The blue boxes represent days with available MODIS images, and the percentage (%) of the study area covered by snow is indicated above the figure. The red dashed line on the relative humidity chart indicates the threshold (95%) above which we estimate a high risk of precipitation and on the air temperature chart it indicated 0°C.

Winter 2014-2015

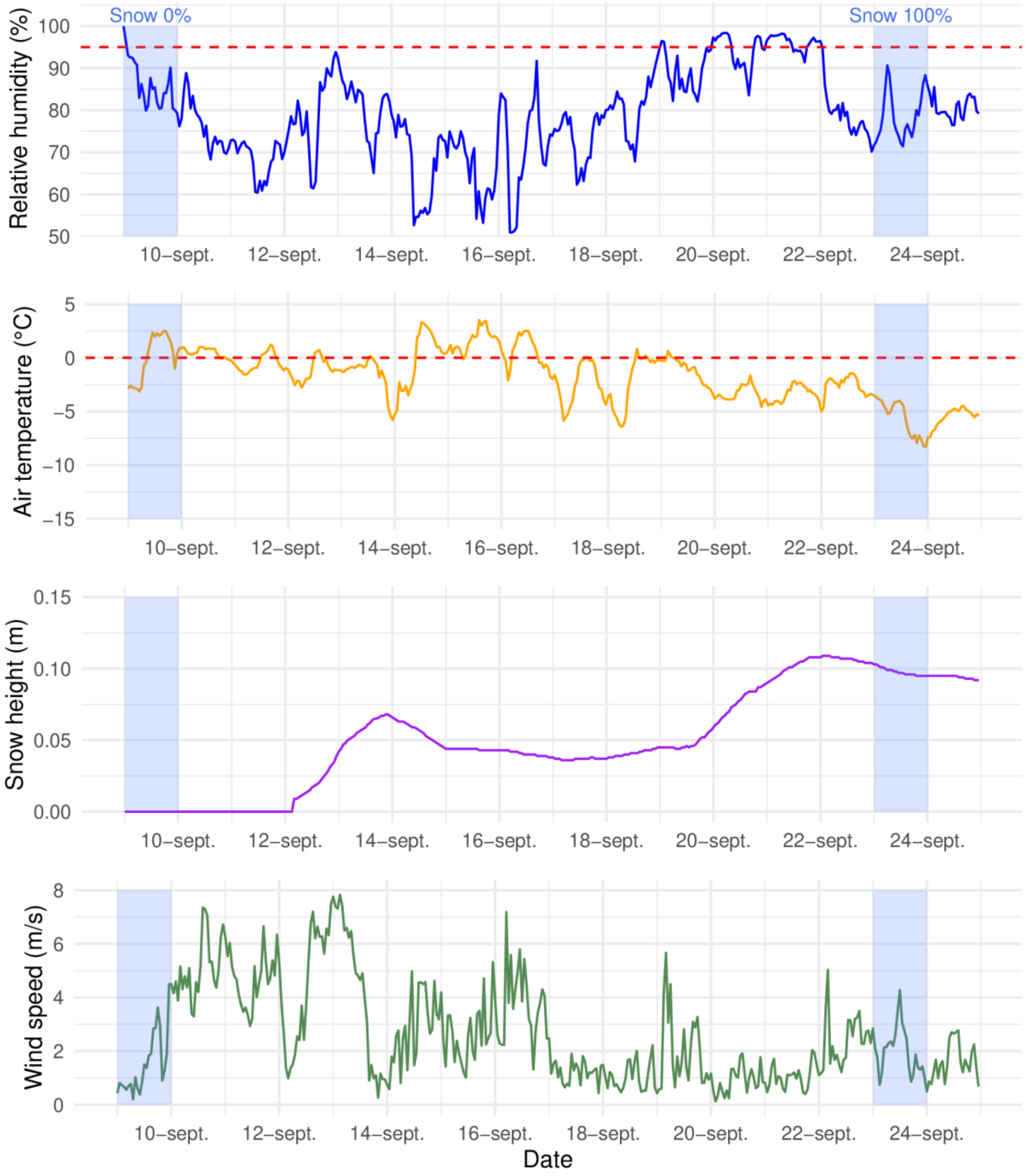

Figure S15. Weather conditions recorded during the period of snow onset in fall 2014. The blue boxes represent days with available MODIS images, and the percentage (%) of the study area covered by snow is indicated above the figure. The red dashed line on the relative humidity chart indicates the threshold (95%) above which we estimate a high risk of precipitation and on the air temperature chart it indicated 0°C.

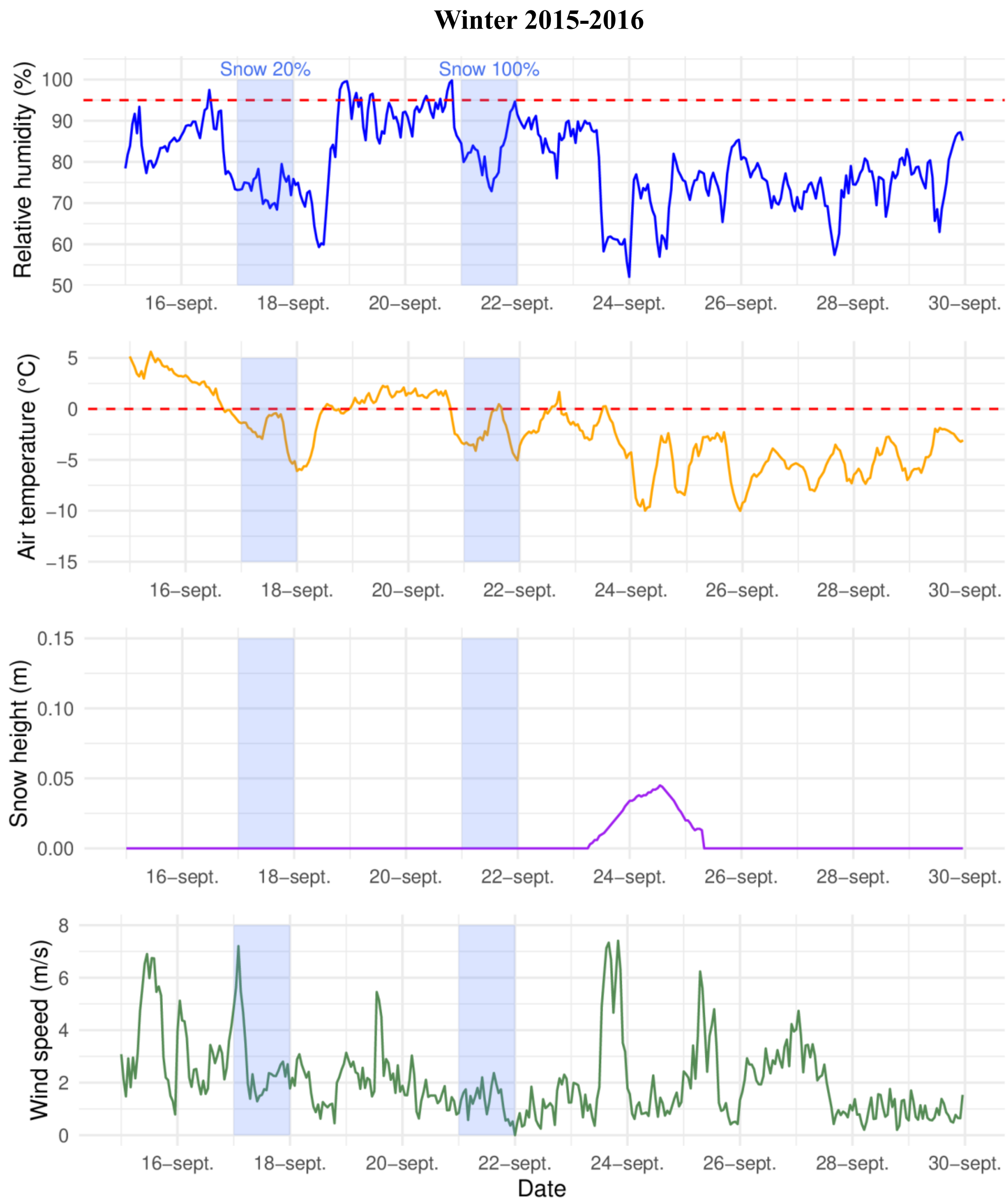

Figure S16. Weather conditions recorded during the period of snow onset in fall 2015. The blue boxes represent days with available MODIS images, and the percentage (%) of the study area covered by snow is indicated above the figure. The red dashed line on the relative humidity chart indicates the threshold (95%) above which we estimate a high risk of precipitation and on the air temperature chart it indicated 0°C.

Winter 2016-2017

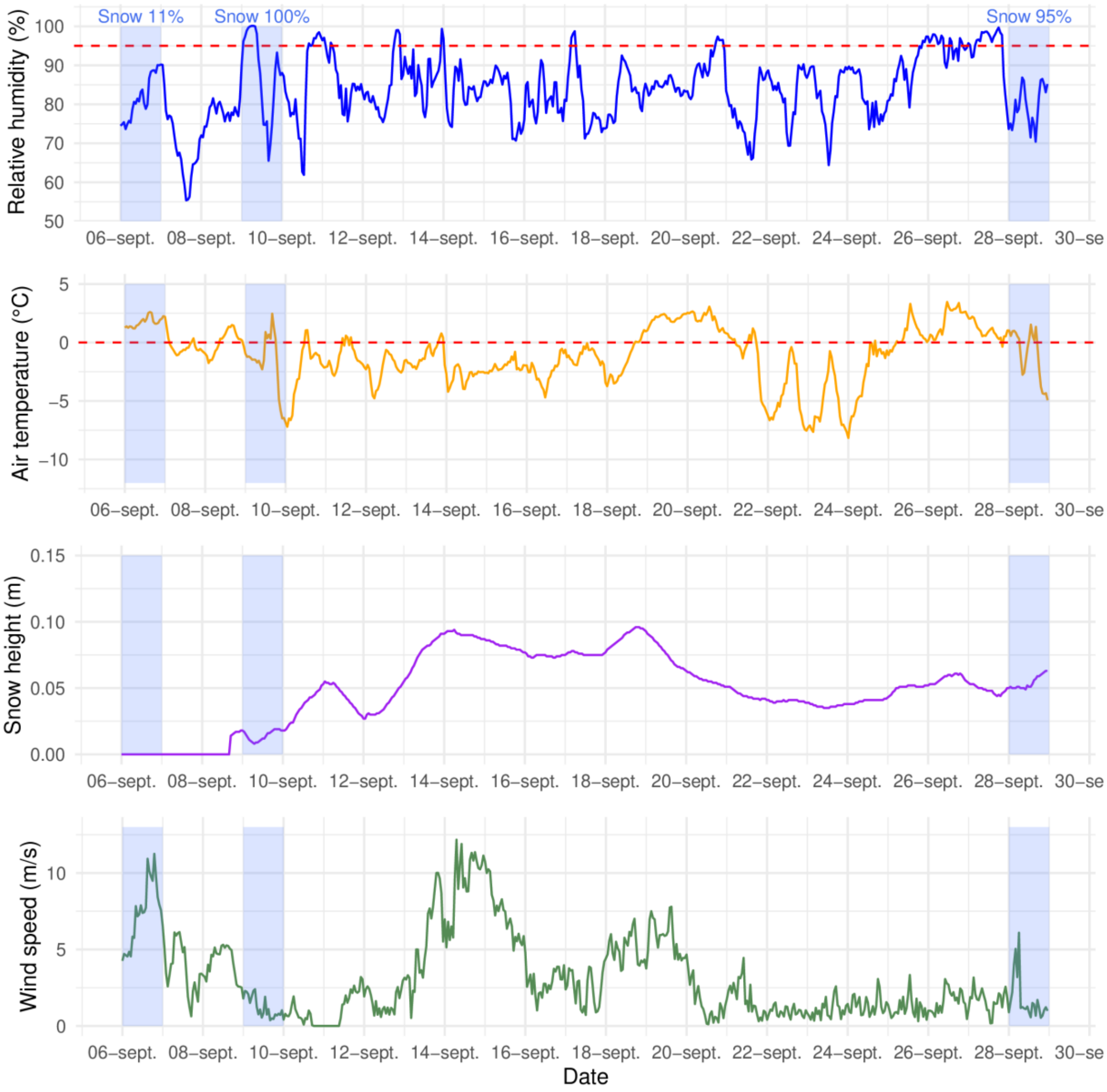

Figure S17. Weather conditions recorded during the period of snow onset in fall 2016. The blue boxes represent days with available MODIS images, and the percentage (%) of the study area covered by snow is indicated above the figure. The red dashed line on the relative humidity chart indicates the threshold (95%) above which we estimate a high risk of precipitation and on the air temperature chart it indicated 0°C.

Winter 2017-2018

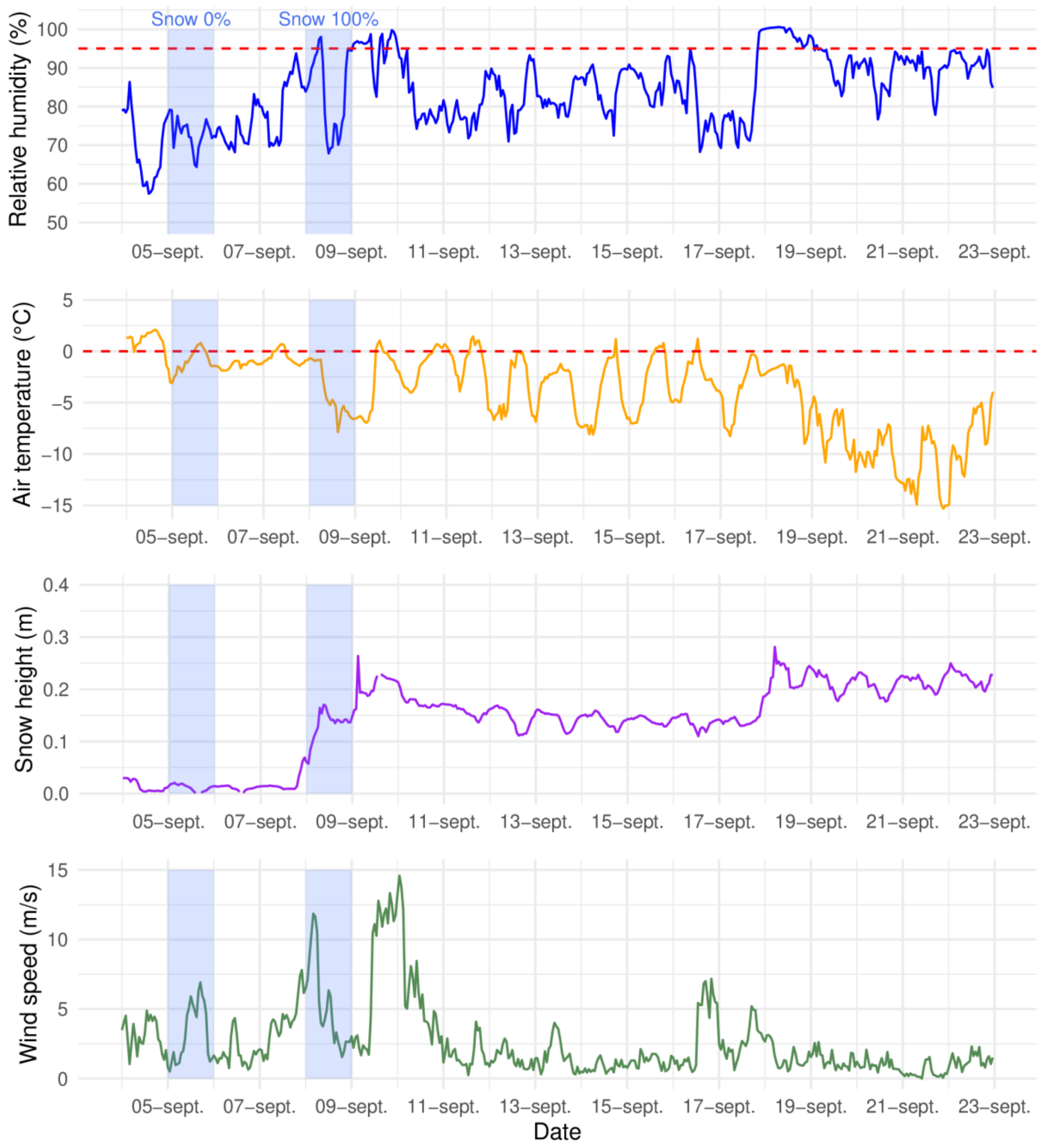

Figure S18. Weather conditions recorded during the period of snow onset in fall 2017. The blue boxes represent days with available MODIS images, and the percentage (%) of the study area covered by snow is indicated above the figure. The red dashed line on the relative humidity chart indicates the threshold (95%) above which we estimate a high risk of precipitation and on the air temperature chart it indicated 0°C.

Winter 2018-2019

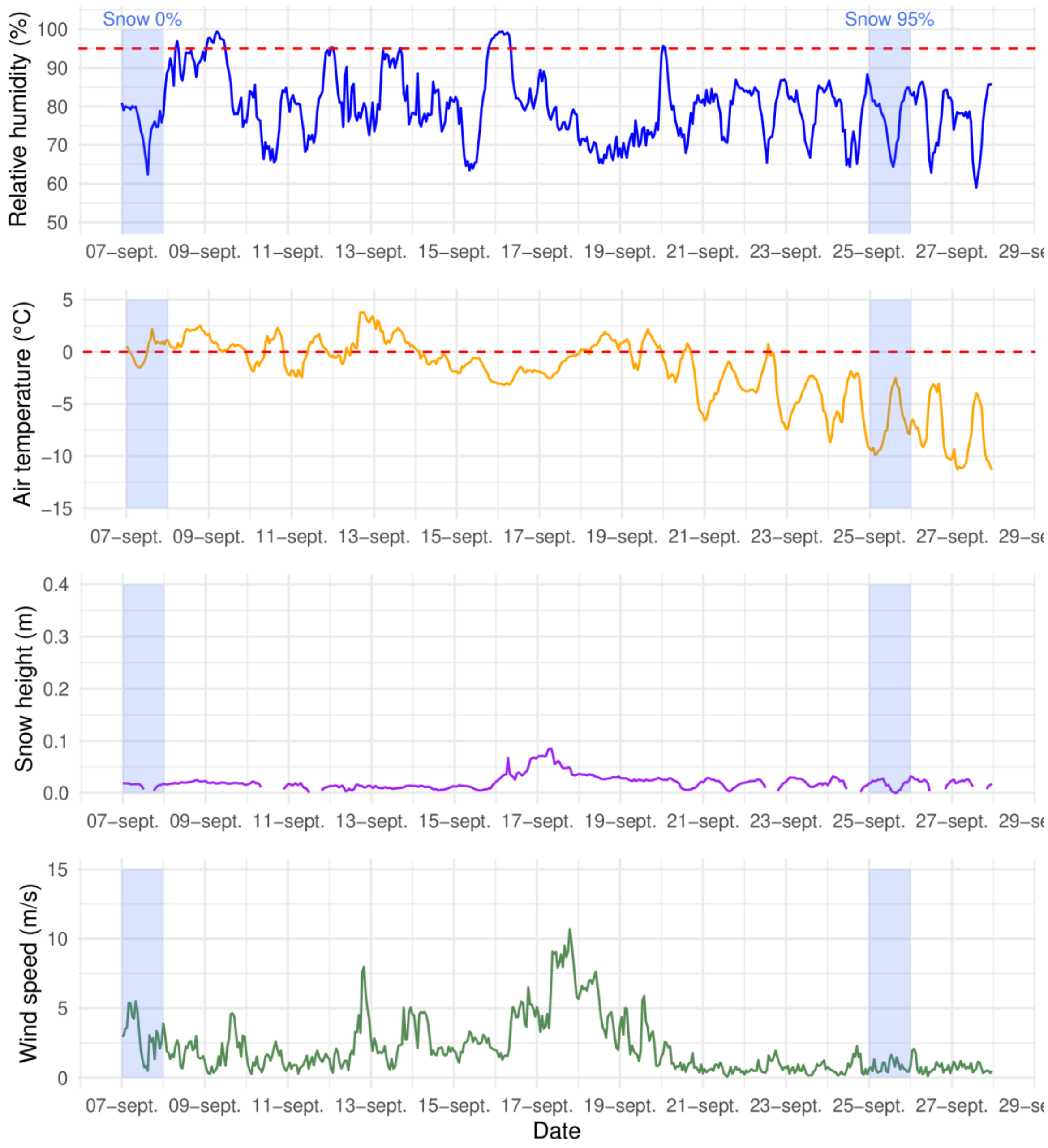

Figure S19. Weather conditions recorded during the period of snow onset in fall 2018. The blue boxes represent days with available MODIS images, and the percentage (%) of the study area covered by snow is indicated above the figure. The red dashed line on the relative humidity chart indicates the threshold (95%) above which we estimate a high risk of precipitation and on the air temperature chart it indicated 0°C.

Winter 2019-2020

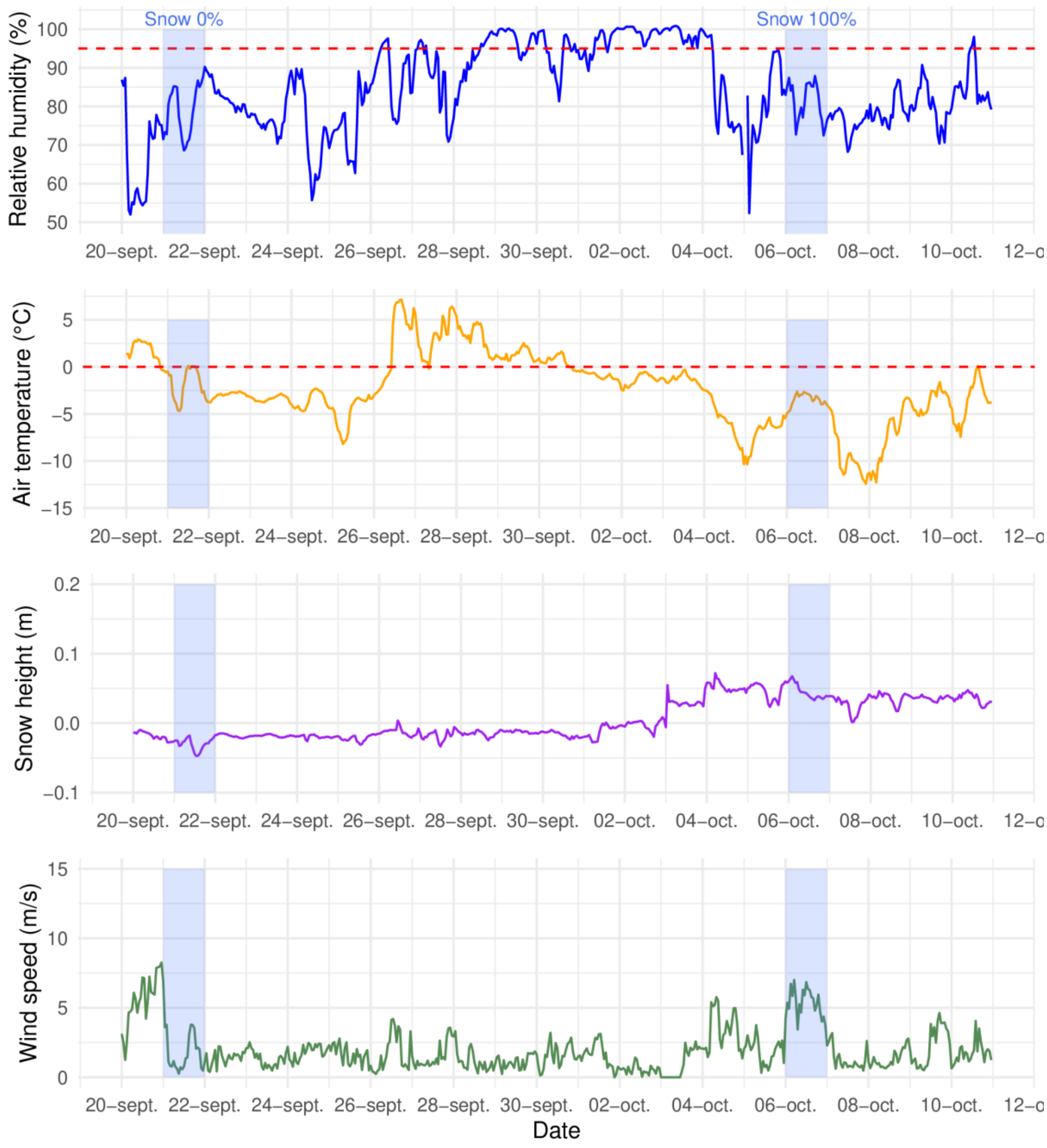

Figure S20. Weather conditions recorded during the period of snow onset in fall 2019. The blue boxes represent days with available MODIS images, and the percentage (%) of the study area covered by snow is indicated above the figure. The red dashed line on the relative humidity chart indicates the threshold (95%) above which we estimate a high risk of precipitation and on the air temperature chart it indicated 0°C.

Winter 2020-2021

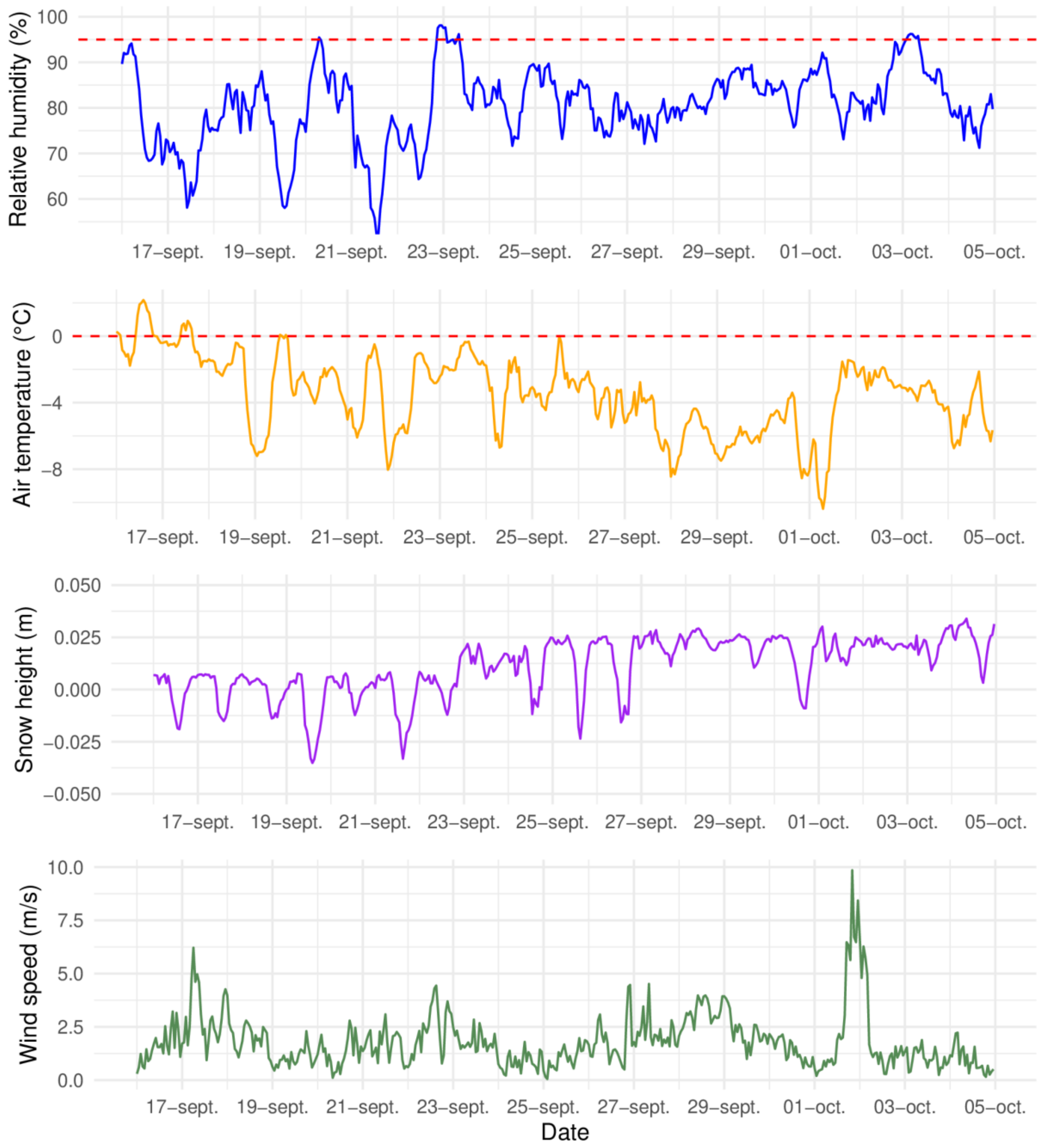

Figure S21. Weather conditions recorded during the period of snow onset in fall 2018. The red dashed line on the relative humidity chart indicates the threshold (95%) above which we estimate a high risk of precipitation and on the air temperature chart it indicated 0°C. MODIS analysis is not available for that year.

Winter 2021-2022

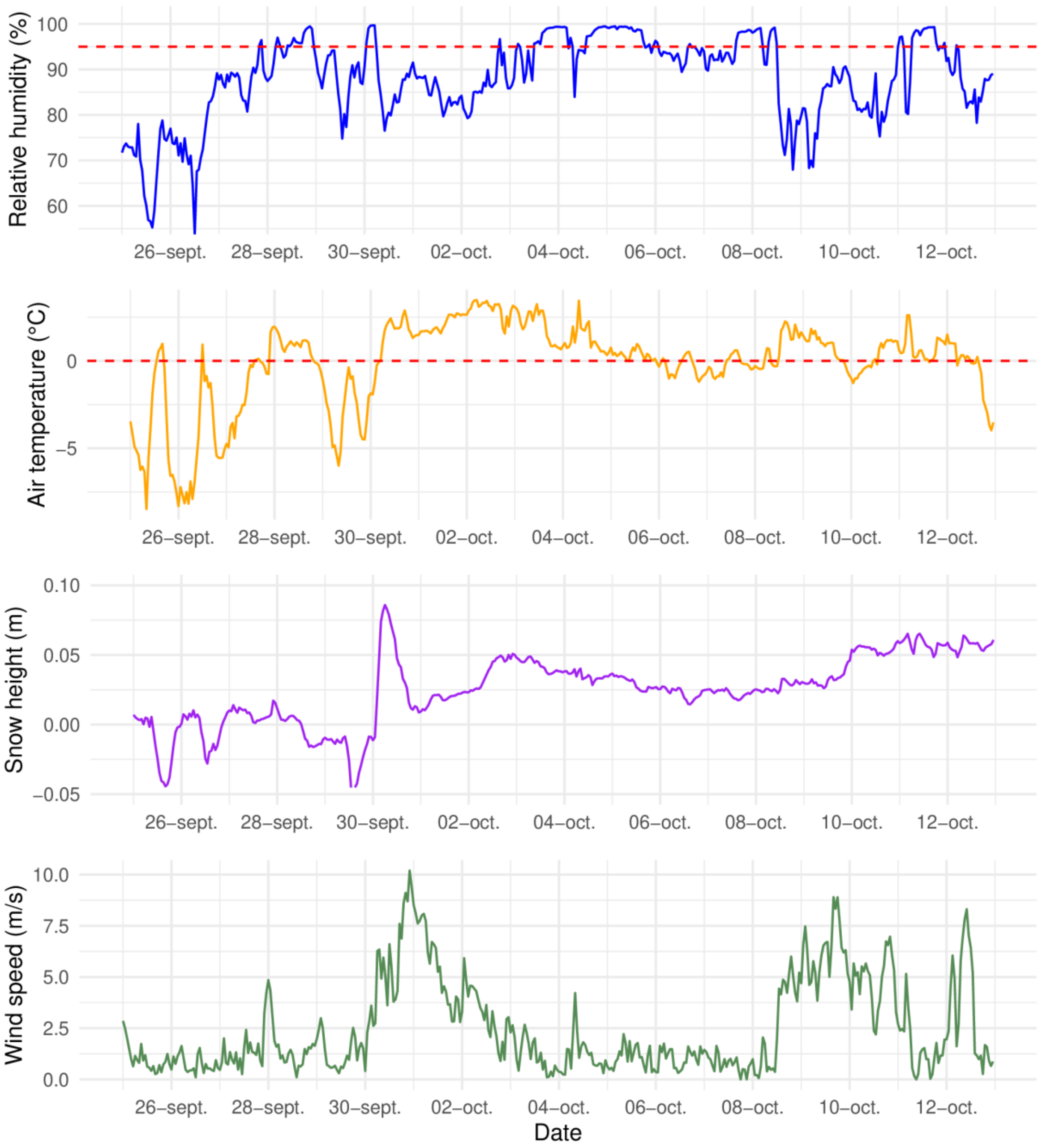

Figure S22. Weather conditions recorded during the period of snow onset in fall 2018. The red dashed line on the relative humidity chart indicates the threshold (95%) above which we estimate a high risk of precipitation and on the air temperature chart it indicated 0°C. MODIS analysis is not available for that year.

References

CEN. 2022. Données des stations climatiques de l'île Bylot au Nunavut, Canada, v. 1.12.0 (1992-2022). Nordicana D2, doi: 10.5885/45039SL-EE76C1BDAADC4890.

Domine, F., G. Lackner, D. Sarrazin, M. Poirier, and M. Belke-Brea. 2021. Données météorologiques, de neige et de sol de l'île Bylot, haut arctique canadien, pour forcer et tester des modèles de neige et de surfaces continentales, v. 1.100000 (2013-2019). Nordicana D86, doi: 10.5885/45693CE-02685A5200DD4C38.

Dozier, J. 1989. Spectral signature of alpine snow cover from the landsat thematic mapper. Remote Sensing of Environment 28:9–22.

Riggs, G., and D. Hall. 2015. MODIS Snow Products Collection 6 User Guide. https://nsidc.org/sites/nsidc.org/files/files/MODIS-snow-user-guide-C6.pdf.
