# Supplementary material for: Demography of lemmings in response to changing snow conditions in the High Arctic
Source: Ecology. 2025 Sep 23;106(9):e70216. doi: 10.1002/ecy.70216 (PMC12457247; doi:10.1002/ecy.70216)
Supplement: Supplementary file 2 — Appendix S2. [file ECY-106-e70216-s002.pdf]

Appendix S2

Demography of lemmings in response to changing snow conditions in the High Arctic

Mathilde Poirier, Gilles Gauthier, Florent Dominé, Dominique Fauteux

Ecology

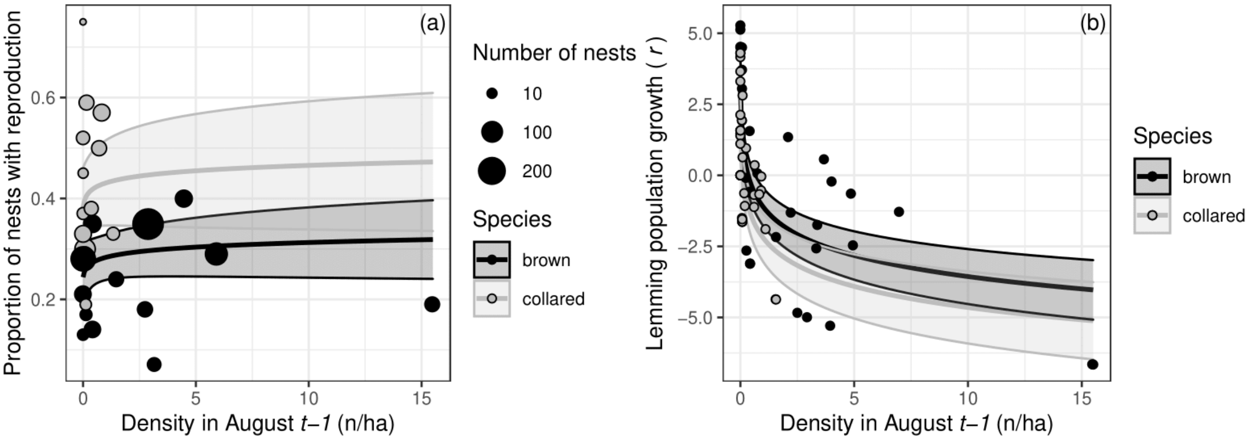

Figure S1. Non-linear relationships between annual proportion of winter nests with reproduction (a) or winter population growth (b) and density in August of the previous year for brown and collared lemmings on Bylot Island, 2004-2022. Values on the x-axis were back-transformed from a logarithmic scale. In (a) size of dots is proportional to sample size. Filled-in areas are 95% CI.

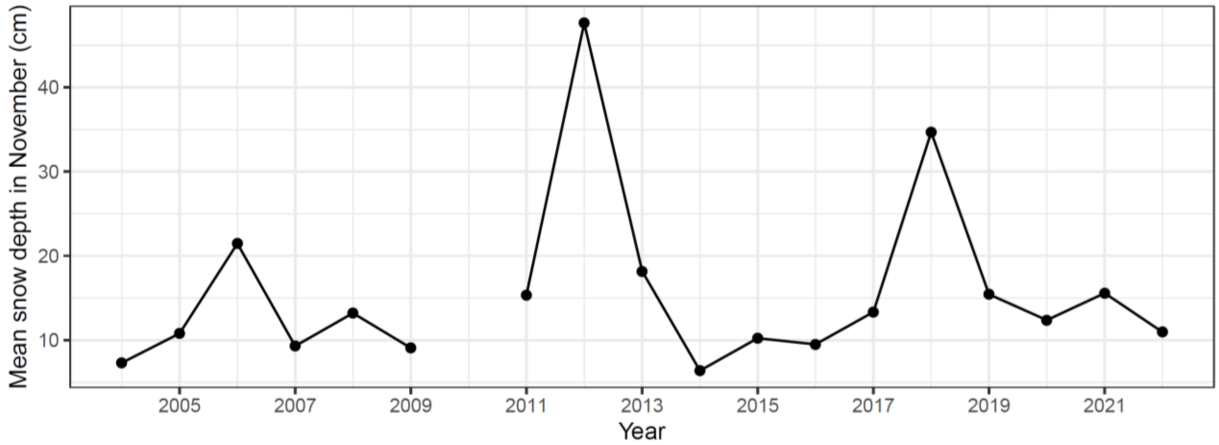

Figure S2. Mean snow depth in November from 2004 to 2022 on Bylot Island. Each winter is referred to by the year when it ended.

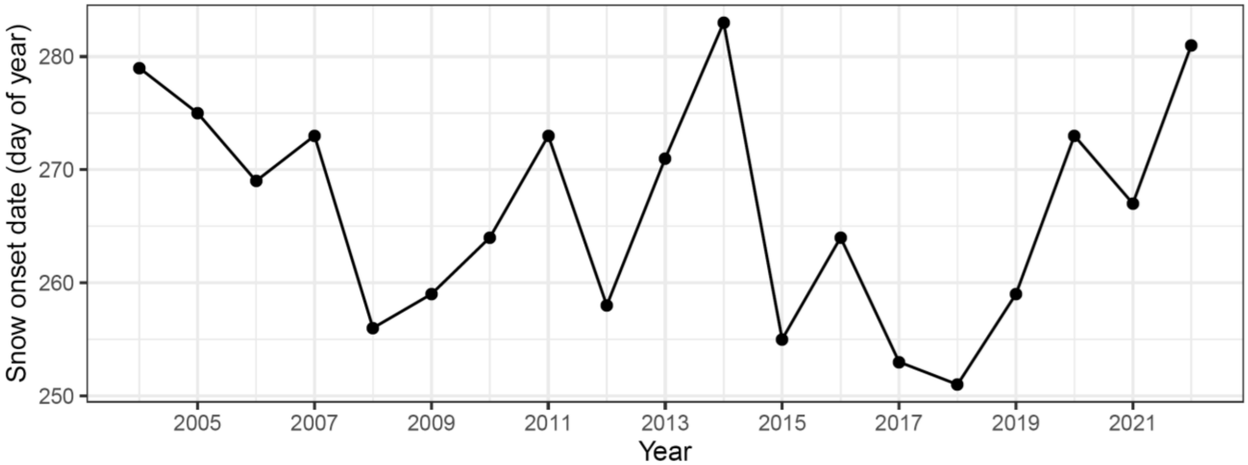

Figure S3. Snow onset date during winters 2004 to 2022 on Bylot Island. Each winter is referred to by the year when it ended.

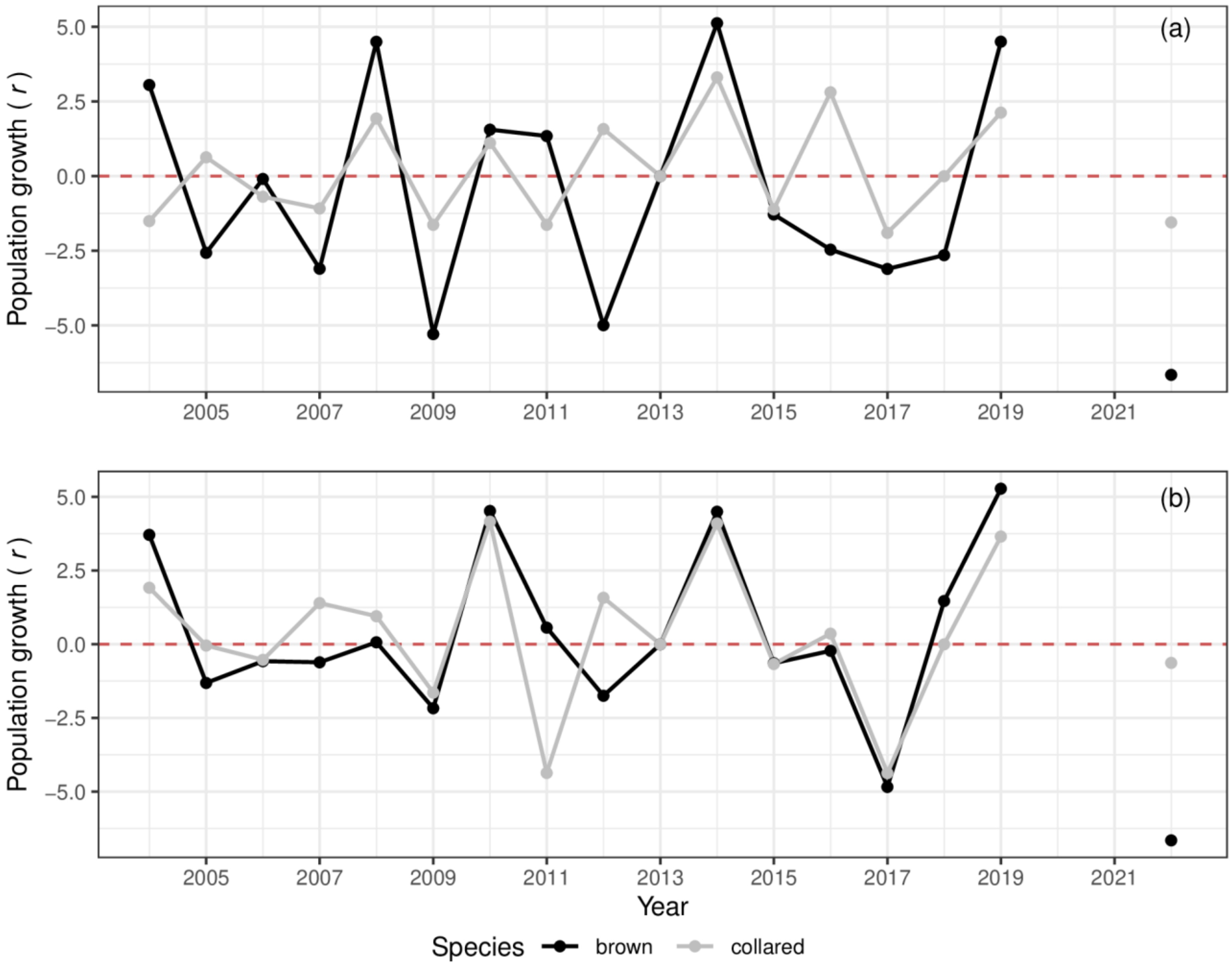

Figure S4. Winter population growth (*r*) of brown and collared lemmings in (a) humid and (b) mesic grids from 2004 to 2022 on Bylot Island. Red line represents a null growth. Each winter is referred to by the year when it ended.

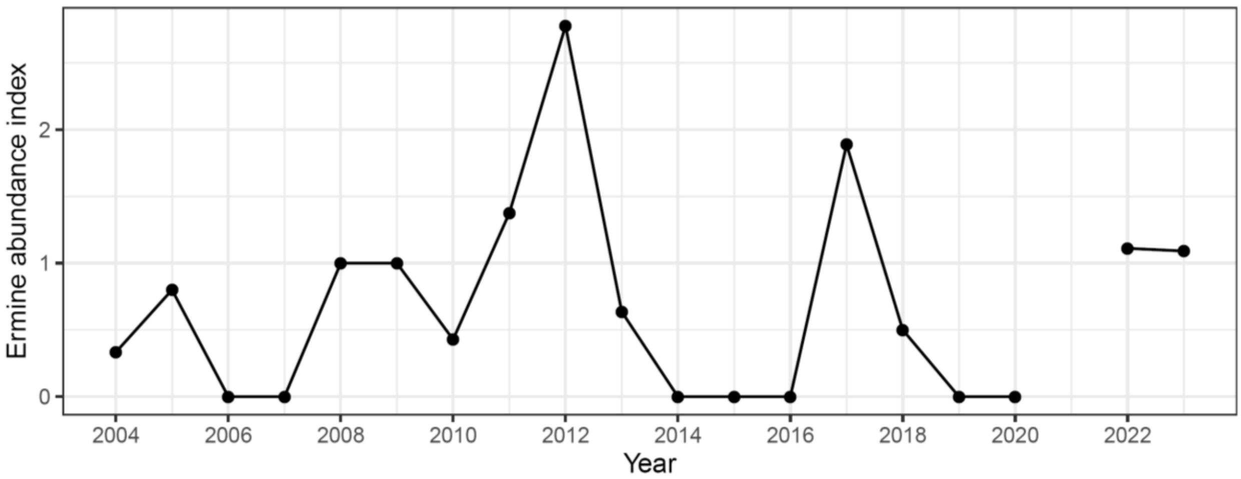

Figure S5. Index of ermine abundance during summers 2004 to 2022 on Bylot Island. Data from (Bolduc et al. 2023).

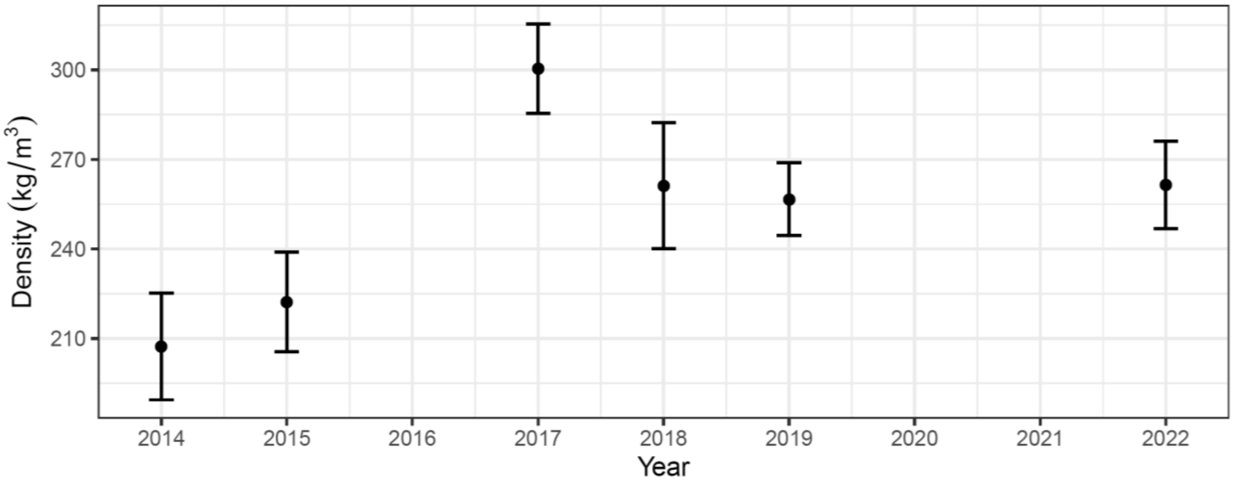

Figure S6. Mean density of the basal snow layer in riparian and mesic habitat during winters 2014 to 2022 on Bylot Island. Error bars represent SE. Each winter is referred to by the year when it ended.

Table S1. Coefficients of all models examining the influence of weather events (rain-on-snow (ros), melt-freeze (melt) and freezing rain (fr)), snow depth in November (depth) and snow onset date (onset) on annual proportion of lemming winter nests with reproduction, with additive or interactive effects of lemming species, density in August of the previous year (density) and ermine abundance of the previous summer on Bylot Island, 2007 – 2022. The slope estimate (*β*), its 95% confidence interval (CI), the number of parameters in the model (*k*), the dispersion parameter (*φ*) and the adjusted R^2 are presented. Models appear in decreasing order of R^2. Conclusive fixed effects are in bold.

Model	Parameter	β	95% CI	φ	k	R2
ros + density + species	(Intercept)	-1.00	[-1.20, -0.76]	1.91	4	0.43
	**log(ros)**	**-0.23**	**[-0.38, -0.08]**			
	log(density)	0.09	[0.00, 0.18]			
	**collared**	**0.76**	**[0.40, 1.12]**			
ros + ermine + species	(Intercept)	-1.16	[-1.44, -1.76]	1.96	4	0.41
	**log(ros)**	**-0.26**	**[-0.42, -0.09]**			
	ermine	0.20	[-0.02, 0.43]			
	**collared**	**0.64**	**[0.31, 0.97]**			
ros*density + species	(Intercept)	-1.01	[-1.22, -0.76]	1.99	5	0.40
	**log(ros)**	**-0.23**	**[-0.39, -0.07]**			
	log(density)	0.10	[0.00, 0.20]			
	**collared**	**0.77**	**[0.39, 1.15]**			
	log(ros)*log(density)	0.02	[-0.08, 0.11]			
ros*species + density	(Intercept)	-1.00	[-1.21, -0.79]	2.26	5	0.40
	**log(ros)**	**-0.22**	**[-0.43, -0.02]**			
	**log(density)**	**0.76**	**[0.37, 1.14]**			
	collared	0.09	[-0.01, 0.19]			
	log(ros)*collared	-0.02	[-0.33, 0.30]			
fr + density + species	(Intercept)	-0.47	[-0.85, -4.76]	2.04	4	0.39
	**log(fr)**	**-0.32**	**[-0.56, -0.09]**			
	log(density)	0.10	[0.00, 0.19]			
	**collared**	**0.80**	**[0.42, 1.17]**			
ros*ermine + species	(Intercept)	-1.19	[-1.50, -1.76]	2.04	5	0.39
	log(ros)	-0.33	[-0.71, 0.06]			
	ermine	0.20	[-0.03, 0.43]			
	**collared**	**0.65**	**[0.31, 0.98]**			
	log(ros)*ermine	0.05	[-0.19, 0.28]			
fr*density + species	(Intercept)	-0.50	[-0.90, -5.76]	2.12	5	0.37
	**log(fr)**	**-0.31**	**[-0.55, -0.07]**			
	log(density)	0.04	[-0.19, 0.28]			
	**collared**	**0.82**	**[0.43, 1.21]**			
	log(fr)*log(density)	0.04	[-0.12, 0.20]			

ros + species	(Intercept)	-1.00	[-1.21, -0.76]	2.12	3	0.36
	**log(ros)**	**-0.20**	**[-0.36, -0.04]**			
	**collared**	**0.60**	**[0.26, 0.94]**			
fr*species + density	(Intercept)	-0.49	[-1.00, 0.02]	2.14	5	0.36
	log(fr)	-0.31	[-0.64, 0.02]			
	**collared**	**0.84**	**[0.04, 1.64]**			
	**log(density)**	**0.10**	**[0.00, 0.20]**			
	log(fr)*collared	-0.03	[-0.49, 0.43]			
melt + density + species	(Intercept)	-0.12	[-0.82, -1.76]	2.16	4	0.35
	**log(melt)**	**-0.24**	**[-0.43, -0.04]**			
	log(density)	0.06	[-0.04, 0.15]			
	**collared**	**0.73**	**[0.34, 1.12]**			
melt + species	(Intercept)	-0.10	[-0.80, -1.76]	2.18	3	0.34
	**log(melt)**	**-0.24**	**[-0.44, -0.05]**			
	**collared**	**0.64**	**[0.29, 1.00]**			
ros*species	(Intercept)	-0.99	[-1.21, -9.76]	2.21	4	0.34
	log(ros)	-0.19	[-0.40, 0.03]			
	**collared**	**0.59**	**[0.24, 0.95]**			
	log(ros)*collared	-0.04	[-0.37, 0.29]			
melt + ermine + species	(Intercept)	-0.12	[-0.88, -1.76]	2.28	4	0.32
	**log(melt)**	**-0.24**	**[-0.45, -0.04]**			
	ermine	0.02	[-0.21, 0.25]			
	**collared**	**0.64**	**[0.28, 1.01]**			
melt*density + species	(Intercept)	-0.15	[-0.87, -1.76]	2.25	5	0.32
	**log(melt)**	**-0.23**	**[-0.43, -0.03]**			
	log(density)	0.14	[-0.35, 0.63]			
	**collared**	**0.72**	**[0.31, 1.12]**			
	log(melt)*log(density)	-0.02	[-0.16, 0.11]			
melt*species + density	(Intercept)	-0.11	[-0.94, 0.72]	2.26	5	0.32
	log(melt)	-0.24	[-0.48, 0.00]			
	collared	0.67	[-1.00, 2.35]			
	log(density)	0.06	[-0.05, 0.16]			
	log(melt)*collared	0.02	[-0.43, 0.46]			
fr + species	(Intercept)	-0.56	[-0.96, -5.76]	2.30	3	0.31
	**log(fr)**	**-0.26**	**[-0.50, -0.02]**			
	**collared**	**0.61**	**[0.26, 0.96]**			
melt*species	(Intercept)	-0.09	[-0.93, -0.76]	2.28	4	0.31

	**log(melt)**	**-0.25**	**[-0.49, -0.01]**			
	collared	0.63	[-1.05, 2.31]			
	log(melt)*collared	0.00	[-0.44, 0.45]			
melt*ermine + species	(Intercept)	-0.59	[-2.42, -5.76]	2.35	5	0.30
	log(melt)	-0.12	[-0.60, 0.37]			
	ermine	0.41	[-0.98, 1.80]			
	**collared**	**0.64**	**[0.27, 1.01]**			
	log(melt)*ermine	-0.10	[-0.46, 0.25]			
fr*species	(Intercept)	-0.61	[-1.13, -6.76]	2.39	4	0.28
	log(fr)	-0.23	[-0.56, 0.11]			
	collared	0.72	[-0.12, 1.56]			
	log(fr)*collared	-0.07	[-0.56, 0.42]			
fr + ermine + species	(Intercept)	-0.58	[-1.04, -5.76]	2.40	4	0.28
	**log(fr)**	**-0.26**	**[-0.50, -0.01]**			
	ermine	0.03	[-0.20, 0.25]			
	**collared**	**0.61**	**[0.26, 0.97]**			
fr*ermine + species	(Intercept)	-0.48	[-1.09, -4.76]	2.48	5	0.26
	log(fr)	-0.32	[-0.67, 0.04]			
	ermine	-0.08	[-0.59, 0.42]			
	**collared**	**0.62**	**[0.25, 0.99]**			
	log(fr)*ermine	0.07	[-0.21, 0.35]			
depth*species + density	(Intercept)	-0.59	[-1.09, -0.10]	2.53	5	0.24
	depth	-0.03	[-0.06, 0.01]			
	collared	0.23	[-0.51, 0.97]			
	log(density)	0.08	[-0.03, 0.19]			
	depth*species	0.03	[-0.02, 0.08]			
depth + density + species	(Intercept)	-0.80	[-1.18, -0.41]	2.63	4	0.21
	depth	-0.01	[-0.03, 0.01]			
	log(density)	0.07	[-0.04, 0.17]			
	**collared**	**0.66**	**[0.24, 1.07]**			
depth + ermine + species	(Intercept)	-0.80	[-1.18, -0.41]	2.64	4	0.21
	depth	-0.02	[-0.05, 0.01]			
	ermine	0.18	[-0.13, 0.49]			
	**collared**	**0.58**	**[0.20, 0.95]**			
depth + species	(Intercept)	-0.81	[-1.20, -0.43]	2.67	3	0.20
	depth	-0.01	[-0.03, 0.01]			
	**collared**	**0.55**	**[0.17, 0.92]**			
onset + density + species	(Intercept)	-2.50	[-7.50, -5.76]	2.66	4	0.20

	onset	0.01	[-0.01, 0.02]			
	log(density)	0.07	[-0.04, 0.18]			
	**collared**	**0.68**	**[0.25, 1.11]**			
depth*species	(Intercept)	-0.66	[-1.15, -0.17]	2.66	4	0.20
	depth	-0.02	[-0.06, 0.01]			
	collared	0.20	[-0.55, 0.95]			
	depth*collared	0.03	[-0.02, 0.08]			
depth*ermine + species	(Intercept)	-1.00	[-1.61, -0.40]	2.68	5	0.20
	depth	0.00	[-0.05, 0.05]			
	ermine	0.30	[-0.12, 0.72]			
	**collared**	**0.57**	**[0.19, 0.96]**			
	depth*ermine	-0.01	[-0.03, 0.01]			
depth*density + species	(Intercept)	-0.73	[-1.19, -0.26]	2.71	5	0.19
	depth	-0.01	[-0.05, 0.02]			
	log(density)	0.11	[-0.08, 0.30]			
	collared	0.63	[0.21, 1.06]			
	depth*collared	0.00	[-0.02, 0.01]			
onset*ermine + species	(Intercept)	0.97	[-5.17, -9.76]	2.69	5	0.19
	onset	-0.01	[-0.03, 0.02]			
	ermine	-5.02	[-11.75, 1.71]			
	**collared**	**0.59**	**[0.21, 0.98]**			
	onset*ermine	0.02	[-0.01, 0.04]			
onset + species	(Intercept)	-1.60	[-6.45, -6.76]	2.73	3	0.18
	onset	0.00	[-0.02, 0.02]			
	**collared**	**0.55**	**[0.17, 0.93]**			
onset*species + density	(Intercept)	-3.71	[-9.93, 2.5]	2.73	5	0.18
	onset	0.01	[-0.93, 0.0]			
	collared	4.27	[-2.93, 14.8]			
	log(density)	0.08	[-0.93, 0.10]			
	onset*collared	-0.01	[-0.93, 0.00]			
onset*density + species	(Intercept)	-2.31	[-7.84, -3.76]	2.78	5	0.17
	onset	0.01	[-0.02, 0.03]			
	log(density)	0.30	[-2.21, 2.80]			
	**collared**	**0.69**	**[0.24, 1.14]**			
	onset*log(density)	0.00	[-0.01, 0.01]			
onset*species	(Intercept)	-2.41	[-8.36, -4.76]	2.82	4	0.15
	onset	0.01	[-0.02, 0.03]			
	collared	3.10	[-7.48, 13.67]			
	onset*collared	-0.01	[-0.05, 0.03]			

onset + ermine + species	(Intercept)	-1.79	[-6.79, -7.76]	2.83	4	0.15
	onset	0.00	[-0.02, 0.02]			
	ermine	0.05	[-0.19, 0.30]			
	**collared**	**0.56**	**[0.17, 0.95]**			

Table S2. Coefficients of models examining the influence of rain-on-snow (ros) or melt-freeze (melt) events on annual proportion of lemming winter nests with reproduction, with and without the inclusion of moderately influential years based on the Cook's distance. For each year excluded, we provide the range of Cook's distance obtained. Main models were selected from Table 1 of the original manuscript. Conclusive fixed effects are in bold.

(a) Models with rain-on-snow (Main model: ros + density + species)

Years excluded	Cook's distance	Parameter	β	95% CI
None	NA	(Intercept)	-1.00	[-1.20, -0.76]
		**log(ros)**	**-0.23**	**[-0.38, -0.08]**
		log(density)	0.09	[0.00, 0.18]
		**collared**	**0.76**	**[0.40, 1.12]**
2014	0.02-0.46	(Intercept)	-1.09	[-1.32, -0.85]
		**log(ros)**	**-0.22**	**[-0.37, -0.07]**
		**log(density)**	**0.16**	**[0.03, 0.29]**
		**collared**	**0.92**	**[0.47, 1.37]**
2017	0.05-0.21	(Intercept)	-1.10	[-1.32, -0.88]
		**log(ros)**	**-0.38**	**[-0.61, -0.16]**
		log(density)	0.09	[0.00, 0.18]
		**collared**	**0.80**	**[0.44, 1.16]**
2011	0.09-0.17	(Intercept)	-1.07	[-1.31, -0.83]
		**log(ros)**	**-0.19**	**[-0.35, -0.03]**
		log(density)	0.05	[-0.05, 0.16]
		**collared**	**0.70**	**[0.30, 1.09]**
2022	0.0005-0.008	(Intercept)	-0.99	[-1.21, -0.77]
		**log(ros)**	**-0.21**	**[-0.39, -0.03]**
		log(density)	0.09	[-0.01, 0.19]
		**collared**	**0.76**	**[0.38, 1.14]**
2017 and 2022	0.0005-0.21	(Intercept)	-1.22	[-1.51, -0.92]
		**log(ros)**	**-0.55**	**[-0.91, -0.20]**
		log(density)	0.08	[-0.01, 0.17]
		**collared**	**0.82**	**[0.46, 1.19]**

(b) Models with melt-freeze (Main model: melt + density + species)

Years excluded	Cook' distance	Parameter	β	95% CI
None	NA	(Intercept)	-0.12	[-0.82, -1.76]
		**log(melt)**	**-0.24**	**[-0.43, -0.04]**
		log(density)	0.06	[-0.04, 0.15]
		**collared**	**0.73**	**[0.34, 1.12]**

2008	0.16-0.17	(Intercept)	0.22	[-0.38, 0.82]
		**log(melt)**	**-0.35**	**[-0.53, -0.18]**
		log(density)	0.06	[-0.02, 0.14]
		**collared**	**0.75**	**[0.41, 1.08]**
2007	0.03-0.15	(Intercept)	-0.19	[-0.84, 0.46]
		**log(melt)**	**-0.21**	**[-0.39, -0.02]**
		log(density)	0.05	[-0.04, 0.14]
		**collared**	**0.78**	**[0.39, 1.17]**
2014	0.01-0.15	(Intercept)	-0.28	[-1.06, 0.50]
		log(melt)	-0.21	[-0.41, 0.00]
		log(density)	0.10	[-0.04, 0.25]
		**collared**	**0.82**	**[0.33, 1.31]**
2017	0.001-0.003	(Intercept)	-0.13	[-0.88, 0.62]
		**log(melt)**	**-0.24**	**[-0.45, -0.02]**
		log(density)	0.06	[-0.05, 0.16]
		**collared**	**0.75**	**[0.33, 1.17]**
2022	0.01-0.02	(Intercept)	-0.23	[-0.96, 0.50]
		log(melt)	-0.20	[-0.41, 0.01]
		log(density)	0.06	[-0.04, 0.17]
		**collared**	**0.74**	**[0.35, 1.14]**
2017 and 2022	0.001-0.02	(Intercept)	-0.26	[-1.07, 0.55]
		log(melt)	-0.19	[-0.43, 0.04]
		log(density)	0.07	[-0.04, 0.18]
		**collared**	**0.76**	**[0.34, 1.19]**

Table S3. Coefficients of all models examining the influence of weather events (rain-on-snow (ros), melt-freeze (melt) and freezing rain (fr)), snow depth in November (depth) and snow onset date (onset)) on winter population growth of lemmings, with additive or interactive effects of lemming species, density in August of the previous year (density) and ermine abundance of the previous summer on Bylot Island, 2007 – 2022. The slope estimate (*β*), its 95% confidence interval (CI), the number of parameters in the model (*k*), and both R^2_m and R^2_c are presented. Models appear in decreasing order R^2_c. Conclusive fixed effects are in bold.

Model	Parameter	*β*	95% CI	*k*	R^2_m	R^2_c
ros*species + density	(Intercept)	-0.70	[-1.66, 0.26]	7	0.62	0.85
	**ros**	**-0.10**	**[-0.18, -0.01]**			
	**collared**	**-1.38**	**[-2.36, -0.41]**			
	**log(density)**	**-1.08**	**[-1.32, -0.83]**			
	**ros*collared**	**0.07**	**[0.01, 0.14]**			
melt*species + density	(Intercept)	0.13	[-1.18, 1.44]	7	0.60	0.85
	**melt**	**-0.02**	**[-0.05, 0.00]**			
	**collared**	**-2.04**	**[-3.41, -0.67]**			
	**log(density)**	**-1.04**	**[-1.29, -0.79]**			
	**melt*collared**	**0.02**	**[0.00, 0.04]**			
fr*species + density	(Intercept)	-0.91	[-2.16, 0.34]	7	0.56	0.84
	fr	-0.03	[-0.12, 0.06]			
	**collared**	**-1.57**	**[-2.62, -0.51]**			
	**log(density)**	**-1.16**	**[-1.43, -0.89]**			
	fr*collared	0.05	[-0.02, 0.11]			
ros + species + density	(Intercept)	-0.85	[-1.80, 0.11]	6	0.61	0.83
	ros	-0.06	[-0.11, -0.01]			
	**collared**	**-1.09**	**[-2.04, -0.14]**			
	**log(density)**	**-1.07**	**[-1.34, -0.81]**			
melt + species + density	(Intercept)	-0.44	[-1.46, 0.59]	6	0.59	0.83
	melt	-0.01	[-0.03, 0.00]			
	**collared**	**-1.11**	**[-2.08, -0.15]**			
	**log(density)**	**-1.09**	**[-1.35, -0.83]**			
ros*log(density) + species	(Intercept)	-0.70	[-1.65, 0.24]	7	0.60	0.83
	ros	-0.06	[-0.12, -0.01]			
	**log(density)**	**-0.98**	**[-1.27, -0.70]**			
	**collared**	**-1.08**	**[-2.04, -0.11]**			
	ros*log(density)	**-0.02**	**[-0.04, 0.00]**			
melt*log(density) + species	(Intercept)	-0.09	[-1.20, 1.02]	7	0.59	0.83
	**melt**	**-0.02**	**[-0.03, 0.00]**			
	**log(density)**	**-0.83**	**[-1.18, -0.48]**			
	**collared**	**-1.10**	**[-2.06, -0.13]**			

	**melt*log(density)**	**0.00**	**[-0.01, 0.00]**			
species + density	(Intercept)	-1.12	[-2.11, -0.14]	5	0.55	0.82
	**collared**	**-1.16**	**[-2.10, -0.22]**			
	**log(density)**	**-1.12**	**[-1.39, -0.85]**			
depth + species + density	(Intercept)	-0.43	[-1.94, 1.09]	6	0.56	0.82
	depth	-0.05	[-0.12, 0.02]			
	**collared**	**-1.13**	**[-2.12, -0.14]**			
	**log(density)**	**-1.14**	**[-1.42, -0.86]**			
onset + species + density	(Intercept)	-5.45	[-27.74, 16.83]	6	0.55	0.82
	onset	0.02	[-0.07, 0.10]			
	**collared**	**-1.16**	**[-2.10, -0.22]**			
	**log(density)**	**-1.12**	**[-1.40, -0.84]**			
fr + species + density	(Intercept)	-1.03	[-2.21, 0.15]	6	0.55	0.82
	fr	-0.01	[-0.08, 0.06]			
	**collared**	**-1.15**	**[-2.11, -0.19]**			
	**log(density)**	**-1.11**	**[-1.39, -0.83]**			
fr*log(density) + species	(Intercept)	-0.97	[-2.16, 0.22]	7	0.55	0.82
	fr	-0.02	[-0.09, 0.05]			
	**log(density)**	**-1.07**	**[-1.37, -0.77]**			
	**collared**	**-1.15**	**[-2.11, -0.19]**			
	**fr*log(density)**	**-0.01**	**[-0.02, 0.00]**			
depth*log(density) + species	(Intercept)	-0.47	[-2.00, 1.07]	7	0.56	0.82
	depth	-0.05	[-0.11, 0.01]			
	**log(density)**	**-1.17**	**[-1.58, -0.75]**			
	**collared**	**-1.13**	**[-2.12, -0.14]**			
	depth*log(density)	0.00	[-0.01, 0.01]			
onset*log(density) + species	(Intercept)	-3.46	[-33.67, 26.74]	7	0.55	0.82
	onset	0.01	[-0.11, 0.12]			
	log(density)	0.10	[-7.78, 7.99]			
	**collared**	**-1.16**	**[-2.10, -0.21]**			
	onset*log(density)	0.00	[-0.03, 0.03]			
depth*species + log(density)	(Intercept)	-0.30	[-1.92, 1.32]	7	0.56	0.81
	depth	-0.06	[-0.13, 0.01]			
	**collared**	**-1.35**	**[-2.51, -0.20]**			
	**log(density)**	**-1.12**	**[-1.42, -0.82]**			
	depth*collared	0.02	[-0.02, 0.05]			
onset*species + log(density)	(Intercept)	-7.12	[-36.21, 21.96]	7	0.54	0.82
	onset	0.02	[-0.09, 0.13]			

	collared	2.18	[-18.30, 22.66]			
	**log(density)**	**-1.12**	**[-1.41, -0.82]**			
	onset*collared	-0.01	[-0.09, 0.07]			
ros + density	(Intercept)	-1.03	[-1.83, -0.22]	5	0.56	0.78
	**ros**	**-0.07**	**[-0.14, -0.01]**			
	**log(density)**	**-0.86**	**[-1.09, -0.64]**			
ros + ermine + density	(Intercept)	-0.69	[-1.59, 0.22]	6	0.57	0.78
	ros	-0.05	[-0.12, 0.01]			
	ermine	-0.60	[-1.34, 0.14]			
	**log(density)**	**-0.86**	**[-1.09, -0.63]**			
melt + ermine + density	(Intercept)	-0.35	[-1.39, 0.69]	6	0.56	0.78
	melt	-0.01	[-0.03, 0.01]			
	ermine	-0.68	[-1.61, 0.24]			
	**log(density)**	**-0.87**	**[-1.10, -0.63]**			
fr + ermine + density	(Intercept)	-0.58	[-1.69, 0.52]	6	0.54	0.78
	fr	-0.02	[-0.07, 0.04]			
	**ermine**	**-0.90**	**[-1.75, -0.05]**			
	**log(density)**	**-0.87**	**[-1.12, -0.62]**			
ros*ermine + log(density)	(Intercept)	-0.52	[-1.38, 0.34]	7	0.59	0.79
	ros	-0.24	[-0.36, -0.11]			
	ermine	-0.74	[-1.39, -0.10]			
	**log(density)**	**-0.84**	**[-1.07, -0.62]**			
	ros*ermine	0.11	[0.04, 0.18]			
depth*ermine + log(density)	(Intercept)	0.72	[-0.72, 2.16]	7	0.58	0.78
	depth	-0.10	[-0.17, -0.03]			
	**ermine**	**-2.12**	**[-3.53, -0.71]**			
	**log(density)**	**-0.89**	**[-1.13, -0.64]**			
	depth*ermine	0.06	[0.02, 0.10]			
melt*ermine + log(density)	(Intercept)	0.34	[-1.30, 1.98]	7	0.56	0.79
	melt	-0.02	[-0.06, 0.02]			
	ermine	-1.66	[-4.05, 0.73]			
	**log(density)**	**-0.86**	**[-1.10, -0.61]**			
	melt*ermine	0.01	[-0.02, 0.05]			
fr*ermine + log(density)	(Intercept)	-0.69	[-1.99, 0.60]	7	0.53	0.78
	fr	0.00	[-0.09, 0.10]			
	ermine	-0.79	[-1.81, 0.22]			
	**log(density)**	**-0.87**	**[-1.12, -0.62]**			
	fr:herm	-0.02	[-0.08, 0.04]			

onset*ermine + log(density)	(Intercept)	-6.83	[-35.41, 21.76]	7	0.53	0.78
	onset	0.02	[-0.08, 0.13]			
	ermine	7.90	[-24.11, 39.92]			
	**log(density)**	**-0.87**	**[-1.10, -0.63]**			
	onset*ermine	-0.03	[-0.16, 0.09]			
onset + ermine + density	(Intercept)	-1.20	[-23.16, 20.77]	6	0.53	0.78
	onset	0.00	[-0.08, 0.08]			
	ermine	-0.92	[-1.86, 0.02]			
	**log(density)**	**-0.88**	**[-1.12, -0.63]**			
melt + density	(Intercept)	-0.56	[-1.52, 0.40]	5	0.53	0.77
	**melt**	**-0.02**	**[-0.03, 0.00]**			
	**log(density)**	**-0.88**	**[-1.10, -0.66]**			
depth + ermine + density	(Intercept)	-0.69	[-2.35, 0.97]	6	0.53	0.78
	depth	-0.01	[-0.12, 0.09]			
	ermine	-0.79	[-1.98, 0.40]			
	**log(density)**	**-0.89**	**[-1.14, -0.64]**			
fr + density	(Intercept)	-1.19	[-2.30, -0.09]	5	0.47	0.77
	fr	-0.02	[-0.09, 0.05]			
	**log(density)**	**-0.89**	**[-1.14, -0.64]**			
depth + density	(Intercept)	-0.78	[-2.17, 0.60]	5	0.48	0.76
	depth	-0.05	[-0.10, 0.01]			
	**log(density)**	**-0.92**	**[-1.18, -0.67]**			
onset + density	(Intercept)	-6.43	[-30.16, 17.30]	5	0.46	0.76
	onset	0.02	[-0.07, 0.11]			
	**log(density)**	**-0.90**	**[-1.15, -0.65]**			
density	(Intercept)	-1.38	[-2.26, -0.51]	4	0.46	0.76
	**log(density)**	**-0.90**	**[-1.15, -0.66]**			
ros + species	(Intercept)	0.19	[-1.20, 1.58]	5	0.21	0.59
	**ros**	**-0.13**	**[-0.25, -0.01]**			
	collared	0.57	[-0.64, 1.79]			
melt + species	(Intercept)	0.97	[-0.75, 2.69]	5	0.15	0.59
	melt	-0.03	[-0.05, 0.00]			
	collared	0.57	[-0.64, 1.79]			
fr + species	(Intercept)	0.18	[-1.51, 1.88]	5	0.08	0.59
	fr	-0.07	[-0.16, 0.02]			
	collared	0.57	[-0.64, 1.79]			

onset + species	(Intercept)	-8.36	[-45.89, 29.18]	5	0.02	0.59
	onset	0.03	[-0.11, 0.17]			
	collared	0.57	[-0.64, 1.79]			
depth + species	(Intercept)	-0.19	[-2.58, 2.19]	5	0.02	0.58
	depth	-0.02	[-0.08, 0.04]			
	collared	0.63	[-0.65, 1.92]			

Table S4. Coefficients of models examining the influence of rain-on-snow (ros) or melt-freeze (melt) events on winter population growth of lemmings, with and without the inclusion of moderately influential years based on the Cook's distances. For each year excluded, we provide the range of Cook's distance obtained. Main models were selected from Table 2 of the original manuscript. Conclusive fixed effects are in bold.

(a) Models with rain-on-snow (Main model: ros*species + density)

Years excluded	Cook's distance	Parameter	β	95% CI
None	NA	(Intercept)	-0.70	[-1.66, 0.26]
		**ros**	**-0.10**	**[-0.18, -0.01]**
		**collared**	**-1.38**	**[-2.36, -0.41]**
		**log(density)**	**-1.08**	**[-1.32, -0.83]**
		**ros*collared**	**0.07**	**[0.01, 0.14]**
2017	0.002-0.51	(Intercept)	-0.50	[-1.62, 0.62]
		**ros**	**-0.20**	**[-0.39, -0.01]**
		**collared**	**-1.34**	**[-2.05, -0.63]**
		**log(density)**	**-0.99**	**[-1.26, -0.72]**
		**ros*collared**	**0.15**	**[0.00, 0.29]**
2011	0.26-0.46	(Intercept)	-0.94	[-1.85, -0.04]
		**ros**	**-0.09**	**[-0.17, -0.01]**
		**collared**	**-1.09**	**[-1.74, -0.44]**
		**log(density)**	**-1.11**	**[-1.34, -0.88]**
		**ros*collared**	**0.06**	**[0.01, 0.11]**
2022	0.002-0.04	(Intercept)	-0.61	[-1.50, 0.27]
		**ros**	**-0.09**	**[-0.17, 0.00]**
		**collared**	**-1.33**	**[-2.09, -0.58]**
		**log(density)**	**-1.03**	**[-1.30, -0.76]**
		**ros*collared**	**0.07**	**[0.00, 0.13]**
2017 and 2022	0.002-0.51	(Intercept)	-0.40	[-1.40, 0.60]
		ros	-0.30	[-0.87, 0.27]
		**collared**	**-1.41**	**[-2.17, -0.64]**
		**log(density)**	**-0.95**	**[-1.24, -0.65]**
		ros*collared	0.38	[-0.06, 0.83]

(b) Models with melt-freeze (Main model: melt*species + density)

Years excluded	Cook's distance	Parameter	β	95% CI
None	NA	(Intercept)	0.13	[-1.18, 1.44]
		**melt**	**-0.02**	**[-0.05, 0.00]**
		**collared**	**-2.04**	**[-3.41, -0.67]**
		**log(density)**	**-1.04**	**[-1.29, -0.79]**

		**melt*collared**	**0.02**	**[0.00, 0.04]**
2008	0.002-0.64	(Intercept)	0.44	[-0.90, 1.79]
		**melt**	**-0.03**	**[-0.05, -0.02]**
		**collared**	**-2.03**	**[-2.92, -1.15]**
		**log(density)**	**-0.95**	**[-1.18, -0.72]**
		**melt*collared**	**0.02**	**[0.01, 0.04]**
2011	0.21-0.41	(Intercept)	-0.28	[-1.80, 1.24]
		**melt**	**-0.02**	**[-0.04, 0.00]**
		**collared**	**-1.46**	**[-2.29, -0.63]**
		**log(density)**	**-1.09**	**[-1.31, -0.87]**
		**melt*collared**	**0.01**	**[0.00, 0.02]**
2017	0.008-0.23	(Intercept)	0.01	[-1.47, 1.49]
		**melt**	**-0.02**	**[-0.04, 0.00]**
		**collared**	**-1.99**	**[-2.90, -1.08]**
		**log(density)**	**-1.06**	**[-1.31, -0.81]**
		**melt*collared**	**0.02**	**[0.00, 0.03]**
2022	0.009-0.06	(Intercept)	0.03	[-1.42, 1.48]
		**melt**	**-0.02**	**[-0.04, 0.00]**
		**collared**	**-2.06**	**[-3.07, -1.05]**
		**log(density)**	**-1.04**	**[-1.30, -0.79]**
		**melt*collared**	**0.02**	**[0.00, 0.04]**
2017 and 2022	0.008-0.23	(Intercept)	-0.18	[-1.74, 1.37]
		melt	-0.01	[-0.04, 0.01]
		**collared**	**-1.95**	**[-2.99, -0.92]**
		**log(density)**	**-1.04**	**[-1.31, -0.77]**
		**melt*collared**	**0.02**	**[0.00, 0.04]**

References

Bolduc, D., D. Fauteux, C. A. Gagnon, G. Gauthier, J. Bêty, and P. Legagneux. 2023. Testimonials to reconstruct past abundances of wildlife populations. Basic and Applied Ecology 68:23–34.
